# Novel Anti-inflammatory Treatments in Cirrhosis. A Literature-Based Study

**DOI:** 10.3389/fmed.2021.718896

**Published:** 2021-09-23

**Authors:** Thit Mynster Kronborg, Henriette Ytting, Lise Hobolth, Søren Møller, Nina Kimer

**Affiliations:** ^1^Gastro Unit, Medical Division, Amager-Hvidovre University Hospital, Hvidovre, Denmark; ^2^Department of Clinical Physiology and Nuclear Medicine 260, Center for Functional and Diagnostic Imaging and Research, Amager-Hvidovre Hospital, Hvidovre, Denmark; ^3^Department of Clinical Medicine, Faculty of Health Sciences, University of Copenhagen, Copenhagen, Denmark; ^4^Novo Nordisk Foundation Centre for Basic Metabolic Research, Faculty of Health Sciences, University of Copenhagen, Copenhagen, Denmark

**Keywords:** liver cirrhosis, inflammation, treatment, cytokines, anti-oxidation, cirrhosis models

## Abstract

Liver cirrhosis is a disease characterised by multiple complications and a poor prognosis. The prevalence is increasing worldwide. Chronic inflammation is ongoing in liver cirrhosis. No cure for the inflammation is available, and the current treatment of liver cirrhosis is only symptomatic. However, several different medical agents have been suggested as potential healing drugs. The majority are tested in rodents, but few human trials are effectuated. This review focuses on medical agents described in the literature with supposed alleviating and curing effects on liver cirrhosis. Twelve anti-inflammatory, five antioxidative, and three drugs with effects on gut microflora and the LPS pathway were found. Two drugs not categorised by the three former categories were found in addition. In total, 42 rodent studies and seven human trials were found. Promising effects of celecoxib, aspirin, curcumin, kahweol, pentoxifylline, diosmin, statins, emricasan, and silymarin were found in cirrhotic rodent models. Few indices of effects of etanercept, glycyrrhizin arginine salt, and mitoquinone were found. Faecal microbiota transplantation is in increasing searchlight with a supposed potential to alleviate cirrhosis. However, human trials are in demand to verify the findings in this review.

## Introduction

Liver cirrhosis is a chronic disease with increasing prevalence. Its most common aetiologies are alcohol consumption, viral hepatitis, obesity, diabetes mellitus, and metabolic syndrome leading to non-alcoholic steatohepatitis as a part of non-alcoholic fatty liver disease (1). In general, liver cirrhosis results from ongoing fibrosis formation, and further progression leads to portal hypertension, hepatic encephalopathy, and an increased risk of organ failure and hepatocellular carcinoma (HCC), which is associated with high mortality (2).

Chronic inflammation in alcoholic liver disease is mediated by a direct response to alcohol and an indirect inflammatory response to gut microbiota-derived lipopolysaccharide (LPS), leading to a stronger oxidative-inflammatory response (3). With ongoing systemic inflammation, endothelial dysfunction, and fibrogenesis (4) evolve in the liver and are associated with elevated inflammatory cytokines and immune cell activation (5). The inflammation may be caused by translocation over the bowel wall of pathogens or derived pathogen-associated molecular patterns (PAMPs) and damage-associated molecular patterns (DAMPs). These are products of microbial origin produced by pathogens and not by the host. Products from apoptotic cells (6) translocate into the portal and systemic circulation via an impaired intestinal barrier. With a continuous injury, the PAMPs and DAMPs can activate hepatic stellate cells (HSC's) with unwanted adverse effects (6). The HSC's are a source of myofibroblasts and portal fibroblasts, which drive the fibrogenic process (2). When quiescent, HSC's mainly act as vitamin A reserves, but they can abundantly secrete extracellular matrix proteins and different proteinases that elicit unwanted liver architecture remodelling when activated.

Currently, there is no cure for this chronic inflammation in cirrhosis, and treatment mainly focuses on symptomatic relief. When ascites develops in the decompensated stage, diuretics, and albumin infusion improve fluid retention and circulatory function after paracentesis. Non-selective beta-blockers (NSBB's) decreases portal hypertension and are used for long-term treatment as primary and secondary prophylaxis of bleeding from oesophageal varices (7, 8). Hepatic encephalopathy can be reversed using antibiotics and lactulose, as the encephalopathy is often triggered by infections and constipation (9).

There is a reduced incidence of HCC in patients treated with NSBB's (10). The underlying mechanism might be a reduction in bacterial translocation from the gut, which may diminish the portal load of PAMP's and thus the hepatic inflammation.

As hepatic inflammation and neo-angiogenesis are critical drivers in the pathogenesis of HCC, the beta-adrenergic blockade may impede angiogenesis through inhibition of vascular endothelial growth factor production and prevent HCC (11). The preliminary studies need further scientific explorations to support this hypothesis.

Diuretics are the first choice treatment of ascites in decompensated cirrhosis (8, 12). However, side effects of diuretics include fluid- and electrolyte disturbances, dehydration, and renal impairment. Albumin infusion is used to prevent the development of hepatorenal syndrome (HRS) and relieve circulatory disturbances in decompensated cirrhosis (13, 14).

In addition to its osmotic effects, albumin has an immunomodulatory effect (14–16) that is measurable by significantly reduced interleukine-6 (IL-6) response in high dose albumin treatment (14). Thus, albumin may improve survival and prevent complications in decompensated cirrhosis, and its immunomodulatory effects require further exploration as they relate to the prevention of acute-on-chronic liver failure (ACLF).

Both inflammation and oxidative stress are considered key elements in the pathology of cirrhosis. When the liver is injured, it may increase reactive oxygen and nitrogen species (ROS, RNS). These intermediates can induce pro-fibrogenic mechanisms. The oxidative stress causes injury by an alteration of DNA, proteins and lipids, resulting in activation of the hepatic stellate cells; hence one of the triggers of fibrogenesis also elicited by inflammatory pathways. Oxidative stress and inflammation are tightly related and can create a vicious cycle to aggravate liver injuries (17).

Multiple pathways are relevant and interesting when seeking to treat liver cirrhosis. In particular, the chronic and systemic inflammatory and oxidative mechanisms that mediate several complications in cirrhosis suggest that inflammatory cascades are possible targets for the treatment of cirrhosis. The available therapy is inadequate in treating fibrogenesis and liver tissue inflammation, and novel targets and therapies are wanted.

The present review aims to evaluate possible anti-inflammatory agents as potential drug candidates that may alleviate, cure or increase survival among patients with liver cirrhosis.

## Methods

A search of the literature published during the last 10 years was conducted in PubMed and Medline. Titles and abstracts were searched for the following key terms in different combinations: “cirrhosis,” “liver cirrhosis,” “cohort,” “inflammation,” “anti-inflammatory,” “chronic liver disease,” “drugs,” “targets,” “cure.” The complete search strategy is described in Supplementary Table 1. Following agreement among the authors, specific searches were then carried out that include the following terms combined with “liver cirrhosis”:

“silymarin,” “anti-TNF-α,” “curcumin,” “faecal microbiota transplantation,” “enoxaparin,” “etanercept,” “artesunate,” “celecoxib,” “aspirin,” “kahweol,” “mitoquinone,” “glycyrrhizin arginine salt,” “pentoxifylline,” “statin,” “emricasan,” “lanifibranor,” “formyl peptide receptor 2 (WKYMVm),” “tanshinone.”

Inclusion criteria for the studies were their full text being in English, and their design being clinical trials, clinical studies, comparative studies, multi-centre studies, case reports, and observational studies. Interventional studies were considered regardless of whether they had a control group or were blinded. In addition, studies evaluating the safety, efficacy, and therapeutic mechanisms of pharmacological agents with anti-inflammatory effects in humans were included, and studies evaluating inflammatory mechanisms in rodent models were also assessed to support our understanding of immunological mechanisms.

Selected studies using rodent models were considered for inclusion, as the differentiation between fibrosis and cirrhosis differs markedly from that in humans. Hence, mentioning cirrhosis was a criterion in rodent studies. Cell model studies were considered relevant only when combined with human- or rodent studies fulfilling all other search criteria.

Studies with an exclusive focus on fibrosis, steatosis, steatohepatitis, and viral hepatitis without cirrhosis were excluded.

## Results

Literature searches were conducted between the 6th of March and the 16th of May 2021. The initial search strategy resulted in 57,853 general hits, which were subsequently reduced to 1,337 drug search-related hits.

After excluding duplicates and irrelevant papers and following the above-stated in- and exclusion criteria, the abstracts of 351 publications were identified and screened for studies evaluating pharmacological agents' safety, efficacy, and therapeutic mechanisms with anti-inflammatory effects in cirrhosis.

**Figure 1:**
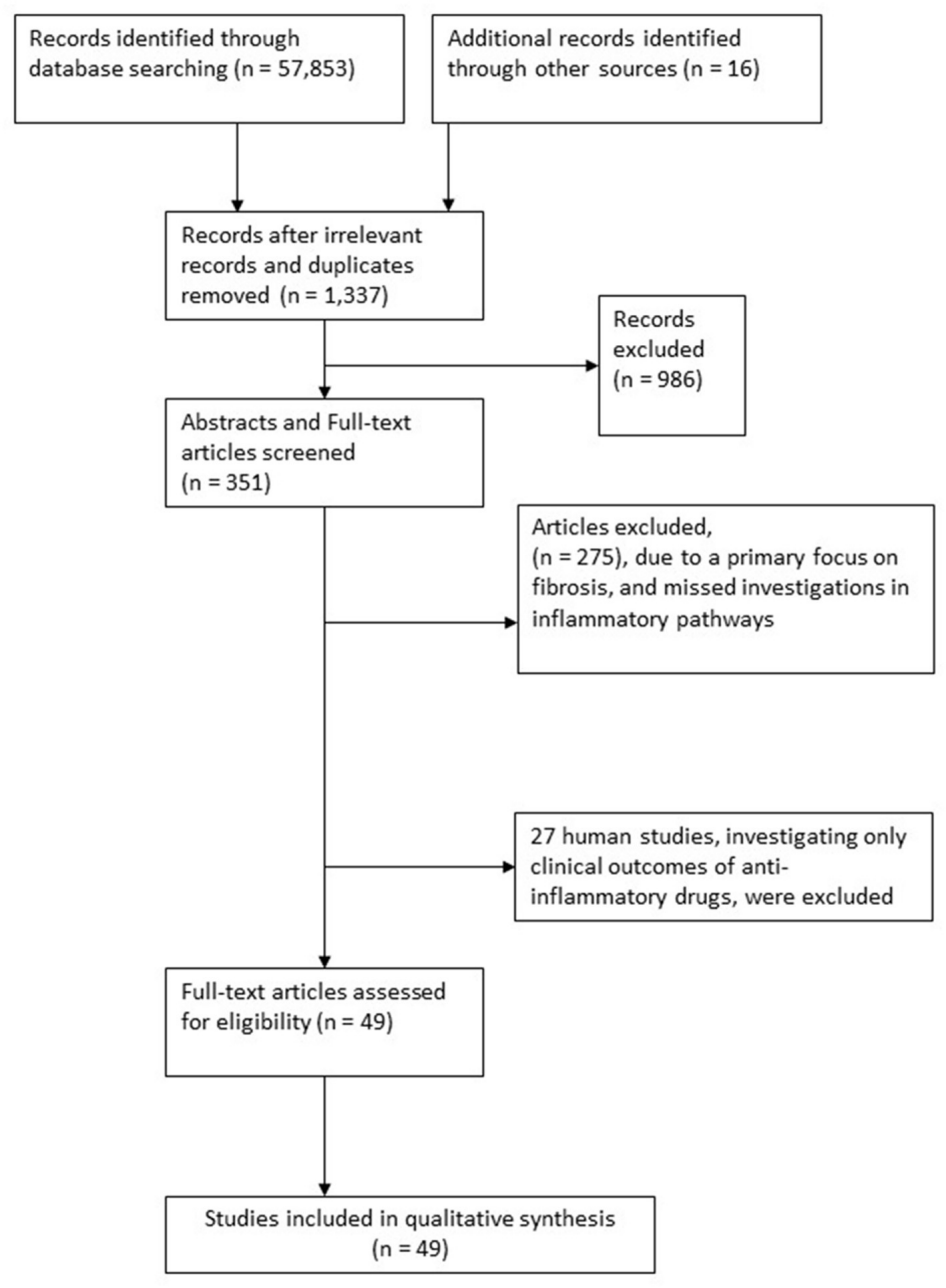
Trial flow chart.

Another 16 papers were found during a manual search of reference lists and bibliographies (Figure 1, Trial flow chart). The remaining 275 papers were excluded due to their primary focus on fibrosis, with no mentioning of cirrhosis or because of a lack of investigations into inflammatory or antioxidative pathways. Seventy-six publications were considered relevant to the research question as evaluated by the authors (SM, NK, and TMK). Of these, 27 studies explored the clinical effects of the included anti-inflammatory drugs in human studies without a particular focus on the anti-inflammatory markers, and these were excluded (see Supplementary Table 2).

Forty-two studies explored potential anti-inflammatory mechanisms of drugs in animal models, and seven studies explored these same drug mechanisms in humans.

Tables 1A,B lists the included studies.

**Table 1A:** Human studies (*N* = 7).

**Study ID**	**Species**	**Intervention**	**Methods**	**Aim**	**Results**
Zafra et al. (18)	Humans	Statins (Simvastatin 40 mg, once 12 h before and once 1 h before the study)	Randomised, double-blind, placebo-controlled trial. 30 patients with liver cirrhosis	Impact on hepatic nitric oxide release and hepatic resistance	Increased hepatosplanchnic output of nitric oxide products. Decreased hepatic resistance
Kaplan et al. (19)	Humans	Statins (Simvastatin 40 mg/day for up to 24 months)	Prospective, multi-centre, double-blind, randomised clinical trial	To investigate the potential reduction of incident hepatic decompensation events among patients at high risk for hepatic decompensation	Not yet available
Frenette et al. (20)	Humans	Emricasan (25 mg twice daily for 3 months, and afterwards 25 mg daily open label)	Multi-centre study, randomised placebo-controlled trial of 86 patients	To investigate the effect of Emricasan on liver function in cirrhosis	Decrease of full-length CK-18 and caspase 3/7. No decrease in cleaved CK-18. Improvement of MELD and Child-Pugh score after 3 months due to improvement in INR and bilirubin
Garcia-Tsao et al. (21)	Humans	Emricasan (25 mg twice daily for 28 days)	Multi-centre, open-label clinical study of 23 patients	Impact on portal hypertension	No significant change in HVPG overall, but sig. decrease in severe PH, AST, ALT, cCK18, and caspase-3/7.
Garcia-Tsao et al. (22)	Humans	Emricasan (5, 25, and 50 mg twice daily for up to 48 weeks)	Multi-centre, double-blinded, randomised clinical study of 263 patients	Testing earlier results of Emricasan decreasing portal hypertension in NASH-related cirrhosis	No significant difference in HVPG for any emricasan dose vs. placebo. Sig. decrease of biomarkers (including Caspase 3/7, cCK18, and flCK18) at week 24, returned to baseline by week 48
Bajaj et al. (23)	Humans	FMT (15 capsules)	Randomised, single-blind, placebo-controlled clinical trial. 20 patients with cirrhosis and recurrent HE	Safety, tolerability, and impact on mucosal/stool microbiota and brain function	Similar episodes of infections and HE in both groups. Reduced LBP in FMT-group. Reduced IL-6 expression post-FMT
Bajaj et al. (24)	Humans	FMT (15 capsules)	Randomised, single-blind, placebo-controlled trial of 20 patients with cirrhosis and recurrent HE	Effect of FMT on the gut-brain axis, inflammation (IL-6 and LPS-binding protein)	Reduced HE-occurrence. Reduced serum IL-6 and LBP

**Table 1B:** Rodent studies (*N* = 42).

**ID**	**Species**	**Drug**	**Methods**	**Aim**	**Results**
Gao et al. (25)	Rats	Celecoxib (20 mg/kg/day)	TAA-induced cirrhosis for 16 weeks during celecoxib administration	Inhibition of COX-2 by celecoxib, reduction of intestinal inflammatory transport	Improvement of intestinal epithelial barrier integrity, blocked inflammatory transport, and diminished progression of cirrhosis
Gao et al. (26)	Rats	Celecoxib (20 mg/kg/day)	TAA-induced cirrhosis for 16 weeks during celecoxib administration	Effect of celecoxib on portal hypertension and the mechanisms behind it.	Dual effects on intrahepatic fibrosis and angiogenesis, Modulation of VEGF/VEGFR-2
Wen et al. (27)	Rats	Celecoxib (20 mg/kg/day)	TAA-induced cirrhosis for 16 weeks during celecoxib administration	Effect on the epithelial-mesenchymal transition of hepatocytes	Amelioration of fibrosis and cirrhosis through suppression of mesenchymal biomarkers. Reduction of intrahepatic inflammation and inhibition of TGF-β1/Smad pathway.
Gao et al. (2)	Rats	Celecoxib (20 mg/kg/day)	TAA-induced cirrhosis for 16 weeks during celecoxib and octreotide administration	Anti-angiogenesis effect of octreotide and celecoxib on cirrhotic portal hypertension	Celecoxib and octreotide relieved fibrogenesis, micro-hepatic arterioportal fistulas, and intrahepatic angiogenesis.
Su et al. (28)	Rats	Celecoxib (20 mg/kg/day)	TAA-induced cirrhosis for 16 weeks during celecoxib administration	To investigate whether celecoxib alleviates liver fibrosis by inhibiting hepatocyte apoptosis via the ER stress response	Celecoxib reduces hepatic apoptosis in TAA-induced cirrhotic rats.
Tang et al. (29)	Rats	Celecoxib (20 mg/kg/day)	TAA-induced cirrhosis for 8 weeks before concomitant continued induction with celecoxib	To examine the impacts of splenomegaly on the development of cirrhosis and assessment of the effects of celecoxib on the splenomegaly and cirrhotic liver.	Celecoxib ameliorates cirrhosis via reducing inflammatory cytokines and immune cells derived from the spleen and suppressing oxidative stress.
Li et al. (30)	Rats	Aspirin (low dose aspirin: 30 mg/kg/day, high dose aspirin: 300 mg/kg/day) and enoxaparin (2 mg/kg/day)	TAA-induced cirrhosis for 4 weeks during aspirin and/or enoxaparin administration	To examine effects of aspirin and enoxaparin in liver function, coagulation index, and histopathology in a rat model of liver fibrosis	Sign. improvement in fibrosis grade in low-dose aspirin, high-dose aspirin, and enoxaparin treated rats.
Assy et al. (31)	Rats	Aspirin (300 mg/kg daily) and enoxaparin (2 mg/kg/day) for 5 weeks	TAA-induced cirrhosis	To examine the effect of aspirin and enoxaparin on fibrosis progression and regenerative activity in a rat model of liver cirrhosis and to determine if the drugs are beneficial in animals with advanced fibrosis or cirrhosis undergoing partial hepatectomy	Sig. improvement in fibrosis grade in both aspirin and enoxaparin group. Improvement of hepatic regenerative activity sig. improved in the aspirin group, unchanged in the enoxaparin group
Abdul-Hamid et al. (32)	Rats	Etanercept (2 mg/kg subcutaneous twice a week for 5 months)	TAA-induced cirrhosis during treatment with etanercept	To clarify the effect of etanercept on the development of cirrhosis and hemosiderosis in rats, highlighting the implication and distribution pattern of hepatic TNF-R1	Diminished expression of hepatic TNF-R1, attenuation of collagen and hemosiderin accumulation, and preservation of hepatic histoarchitecture
Abo-Zaid et al. (33)	Rats	Curcumin (150, 200 or 250 mg/kg/day for 6 weeks)	CCl_4_-induced cirrhosis during curcumin injections	To evaluate the immune regulatory effect of curcumin in hepatic cirrhotic rats	IL-10 sig. increased in curcumin groups, TNF-α and TGF-1β decreased. Curcumin tended to retain the normal structure of liver tissues.
Macías-Pérez et al. (34)	Hamsters	Curcumin (30 mg/kg/day for 4 weeks)	CCl_4_-induced cirrhosis before Curcumin administration	To evaluate reversal of cirrhosis by doxazosin, carvedilol, and curcumin and studying possible modulation of Nrf-2 and NF-kB	a/b adrenergic blockers with curcumin reverse hepatic damage, possibly as a result of adrenergic antagonism on HSC and conceivably by the increase of Nrf-2/NF-kB mRNA ratio
Kyuong et al. (35)	Rats	Curcumin (10 mg/mL orally for 4 weeks)	DMN induced cirrhosis	To investigate the hepatoprotective effect of curcumin. Comparison of curcumin and lactulose treatment in cirrhotic rats	Increased electrical conductivity when treated with curcumin or lactulose compared to the cirrhotic model, sig. levels of attenuated fibrosis and decreased inflammatory response after curcumin and lactulose.
Chenari et al. (36)	Rats	Curcumin (100 mg/kg/day for 4 weeks)	BDL-induced cirrhosis	To explore the hepatoprotective activity of curcumin via measuring expression of SIRT3, AMPK, CPT-1A, IDH2, and MnSOD and lipid profile	Increase of SIRT3, AMPK, CPT-1A, IDH2, and MnSOD and improvement of lipid profile when fed curcumin
Hsu et al. (37)	Rats	Curcumin (600 mg/kg/day for 2 weeks)	CBDL-induced cirrhosis	To evaluate the effects of curcumin as an antiproliferative, anti-inflammatory, and anti-angiogenic agent	Decrease of flow in the superior mesenteric artery and increased resistance. Sig. reduction of eNOS, COX2, VEGF, and pErk. Decrease of portosystemic shunting, induction of vasoconstriction. Amelioration of portal hypertension.
Cai et al. (38)	Rats	Curcumin (200 mg/kg/day for 12 weeks)	CCl_4_-induced cirrhosis	To test the anti-endotoxemia effect of curcumin on induced cirrhosis in rats, elucidate the underlying molecular mechanism.	Improvement of physiological condition, amelioration of liver injury, reduction of inflammatory cytokines in serum and liver tissue, a decrease of LPS in a peripheral vein
Hernández-Aquino et al. (39)	Rats	Curcumin (100 mg/kg twice a day for 3 weeks)	CCl_4_-induced cirrhosis partly before Curcumin administration	To investigate fibrosis reduction in cirrhotic rats and to determine the canonical/non-canonical Smad3 pathways and HSC activation/deactivation induced by curcumin	Reduced liver damage, restoration of levels of MMP-9, MMP-2, Nf-kB, IL-1, IL-10, TGF-β, CTGF, Col-1, MMP-13, Smad-7, α-SMA, and Smad-3. Decrement in hepatic stellate cells
Seo et al. (40)	Mouse liver cell model	Kahweol (doses not specified)	Addition of kahweol in LPS- Kupffer cells and hepatocytes	Effect of kahweol on liver inflammation	Decrease of LPS-induced production of IL-1a, IL-1b, IL-6, and TNF-α. Downregulation of phosphor-NF-kB and—signal transducer and activator of transcription 3 expression.
Arauz et al. (41)	Mice	Kahweol (200 mg/kg twice a day caffeinated or decaffeinated for 8 weeks)	TAA-induced cirrhosis during coffee administration.	Antifibrotic properties of coffee	Blockade of TGF-1β and connective tissue growth factor.
Ali et al. (42)	Rats	Pentoxifylline (100 mg/kg/day) and/or diosmin (50 mg/kg/day) for 28 days	BDL-cirrhotic rats	Effects on inflammatory response oxidative balance, cytoglobin	Downregulation of Keap-1/Nrf-2/GSH and NF-kB-p65/p38-MAPK pathways
Ali et al. (43)	Rats	Diosmin (100 mg/kg/day) and sildenafil (10 mg/kg twice daily) for 4 weeks	BDL-induced cirrhosis	Effects of diosmin on fibrotic markers, oxidation levels, and diverse oxidative markers	Downregulation of NF-kB-p65, P38-MAPK, Keap-1, and iNos.
Tahir et al. (44)	Rats	Diosmin (10 mg/kg or 20 mg/kg for 4 weeks)	Ethanol-induced cirrhosis, diosmin before ethanol (increasing dose of ethanol for 28 days).	Efficacy of diosmin on hepatotoxicity, free radicals, oxidative status, transcription factors, and inflammatory markers.	Diosmin normalised CYP 450 2E1 and alcohol dehydrogenase, attenuated oxidative stress, and alleviated ethanol-induced NF-kB activation as well as TNF-α, COX-2, and iNos.
Zhang et al. (45)	Rats	Glycyrrhizin arginine salt (75 or 150 mg/kg for 2 weeks)	BDL-induced cirrhosis	Effect of glycyrrhizin arginine salt on cirrhosis	Decrease of serum bilirubin, AST, 8-isoprostane and malondialdehyde, Slower fibrogenesis. Reduction of bile salt pool, hydroxyproline, TGF-β1, α -SMA, TNF-α, MMP-2, and MMP-9.
Tripathi et al. (46)	Rats	Statin, 25 mg/kg/day for CCl_4_- and TAA induced cirrhosis, 5 mg/kg/day for BDL cirrhosis (simvastatin for 3 days)	CCl_4_-, BDL, and TAA induced cirrhosis	Effect on ACLF	Prevention of ACLF-complications and improved survival. Reduction of inflammation and oxidation markers
Meireles et al. (47)	Rats	Statin (simvastatin 5 mg/kg/day for 3 days)	BDL induced cirrhosis	Impact of cirrhotic microcirculation and hepatoprotection	Aggravation of microvascular dysfunction and upregulation of inflammatory pathways; prevention of endothelial dysfunction
Uschner et al. (48)	Rats	Statin (atorvastatin 15 mg/kg for 7 days)	BDL + CCl_4_-induced cirrhosis	Investigation of angiogenesis and the hedgehog pathway.	Inhibition of the non-canonical Hh-pathway and angiogenesis.
Shirin et al. (49)	Rats	Statin (atorvastatin 1, 10 or 20 mg/kg/day, rosuvastatin 2.5, 5, 10, or 20 mg/kg/day for 12 weeks)	TAA-induced cirrhosis concomitantly with atorvastatin/rosuvastatin/saline.	Prevention of cirrhosis	No inhibition of cirrhosis or oxidative stress
Jang et al. (50)	Rats	Statin and MSCs (1 × 10^6^ MSC's two times during 12 weeks, and/or 10 mg/kg/day of simvastatin for 5 weeks)	TAA-induced cirrhosis	Synergistic effect of simvastatin and MSCs on fibrosis	Decreased collagen distribution, lowered hydroxyproline content
Gracia-Sancho et al. (51)	Rats	Emricasan (10 mg/kg/day for 7 days)	CCl_4_-induced cirrhosis. *In vitro* experiment on hepatocyte expressions	Effects on haemodynamics, hepatic cells phenotype	Lowered portal pressure, reduced hepatic inflammation, and reduced fibrosis. *In vitro* experiment improved hepatocyte expression
Boyer-Diaz et al. (52)	Rats and human samples	Lanifibranor (100 mg/kg/day for 2 weeks)	TAA-induced cirrhosis for 12 weeks and BDL induced secondary biliary cirrhosis in two separate rat groups. Human samples from liver resections	Therapeutic potential of pan-PPAR activation for the treatment of advanced cirrhosis	For the rats with TAA-cirrhosis: Sig. decrease in portal pressure, reduction of ascites, and cirrhosis regression. Attenuation of the hepatic proinflammatory environment through cytokine expression pattern shift. For human hepatocytes: improvement and amelioration of HSC phenotype and reduction in contraction capacity
Jun et al. (53)	Rats	WKYMVm (2.5 mg/kg twice pr. week for 22 weeks)	BDL-induced cirrhosis	Effects on hepatic regeneration via vascular remodelling, resulting from its pro-angiogenic properties	Improvement of vascular remodelling, inhibition of fibrosis, and enhanced hepatic function
Vilaseca et al. (54)	Rats and human liver cells	Mitoquinone (5 mg/kg/day for 14 days)	HSCs exposed to mitoquinone. CCl_4_- and TAA-induced cirrhosis.	Effects of mitoquinone on hepatic oxidative stress, HSC phenotype, inflammation markers, and liver fibrosis	Decrease of proliferation in both HSCs and rats. Decrease in hepatic oxidative stress and diminished fibrosis
Turkseven et al. (55)	Rats	Mitoquinone (10 mg/kg/day for 25 days)	BDL-induced cirrhosis	Effect on oxidative stress, inflammation markers, fibrosis, and mitophagy.	Prevention of inflammation, hepatocyte necrosis, and fibrosis by mitoquinone. Decrease of TNF- α, TGF-1β, collagen, IL-6, IL-1β, and metalloproteinases. Attenuation of apoptosis by reduced expression of cleaved caspase-3.
Zaidi et al. (56)	Rats	Silymarin vs. saline (200 mg/kg twice a week for 8 weeks)	TAA-induced cirrhosis	Effects of silymarin on liver enzymes, antioxidant enzymes, glutathione reductase, and MDA	Restoration of antioxidant enzymes (SOD and GSH), MDA, and catalase activity
Pour et al. (57)	Rats	Silymarin (50 mg/kg/day) and/or lactulose (2 g/kg/day) for 8 weeks	TAA-induced cirrhosis	Possible synergic and healing effects	Decrease in liver enzymes and malondialdehyde levels
Ali et al. (58)	Rats	Curcumin (400 mg/kg), silybin-phytosome (400 mg/kg), alpha-R-lipoic acid (200 mg/kg/day), or saline. For 7 weeks.	TAA-induced cirrhosis	Protective effects	Blockade of malondialdehyde (MDA) and protein carbonyls. Decrease of GSH-depletion, collagen deposition, MMp-2activity, TGF-1β levels, a-SMA, and HSP-47 expression
Abdel-Moneim et al. (59)	Rats	Silymarin (100 mg/kg five times a week for 4 weeks), taurine (100 mg/kg five times a week for 4 weeks), or both, or olive oil	CCl_4_-induced cirrhosis	Hepatoprotective effect	Alleviation of thiobarbituric acid reactive substances, reduction of NO levels, and NOS activity. Increase of superoxide dismutase, glutathione peroxidase, and glutathione reductase. Reduction of TGF-1β, IL-6, and TNF-α. Combination therapy decreased adiponectin levels and normalised FFA
Aithal et al. (60)	Rats	Silymarin (100 mg/kg) and/or bone-marrow-derived stromal cells (5.8 mill. cells in 0.5 mL) for 3 weeks	CCl_4_-induced cirrhosis	Efficiency and hepatic differentiation potential of BM-MSCs in combination with silymarin	Ameliorated liver tissue damage through immunoregulatory activities. Decrease in liver enzymes and diminished fibrosis. Combination treatment was most efficient compared with individual treatments
Yang et al. (61)	Rats	Tanshinone (10, 20, or 40 mg/kg for 1 week)	CCl_4_- and concomitant alcohol-induced cirrhosis	Investigate therapeutic effects of tanshinone by promoting proliferation and differentiation of stem cells.	Improvement of histology, liver markers, and promotion of proliferation and differentiation of endogenous liver stem cells.
Liu et al. (62)	Germ-free Mice	FMT (0.2 mL daily gavage for 3 days)	CCl_4_-induced cirrhosis in conventional and germ-free mice.	Effect of colonisation using human donors on cortical and liver inflammation markers	Reduced neuroinflammation, and microglial activation and dysbiosis. Liver inflammation was unaffected.
Chen et al. (63)	Rats	Artesunate (25 mg/kg/day for 8 weeks)	CCl_4_-injection and ethanol-induced cirrhosis. Concomitant artesunate or oil solution	Effect of artesunate on bacterial translocation and gut microbiota	Decrease of IL-6 and TNF-α levels. Positive effect on dysbiosis and reduction of bacterial translocation.
Fortea et al. (64)	Rats	Enoxaparin (40 IU/kg/day or 180 IU/kg/day) vs. saline for 12 weeks	CCl_4_-induced cirrhosis, BDL induced cirrhosis.	Effects on advanced cirrhosis	No effect on fibrosis, profibrogenic gene expression, or infection. No amelioration of IL-6 levels. Hepatic arterial dysfunction was corrected.
Cerini et al. (65)	Rats	Enoxaparin (1.8 mg/kg subcutaneously) for 24 and 1 h or daily for 1 week or daily for 3 weeks	CCl_4_-induced cirrhosis, TAA-induced cirrhosis, concomitant Enoxaparin.	Effects on hepatic and systemic haemodynamics, fibrosis, and nitric oxide availability	Decreased portal pressure. Reductions in fibrosis, fibrin deposition, HSC-activation (α-SMA, pro-collagen), and desmin expression

Agents were assessed according to their pathway mechanisms.

### Anti-inflammatory Mediators

**Figure 2:**
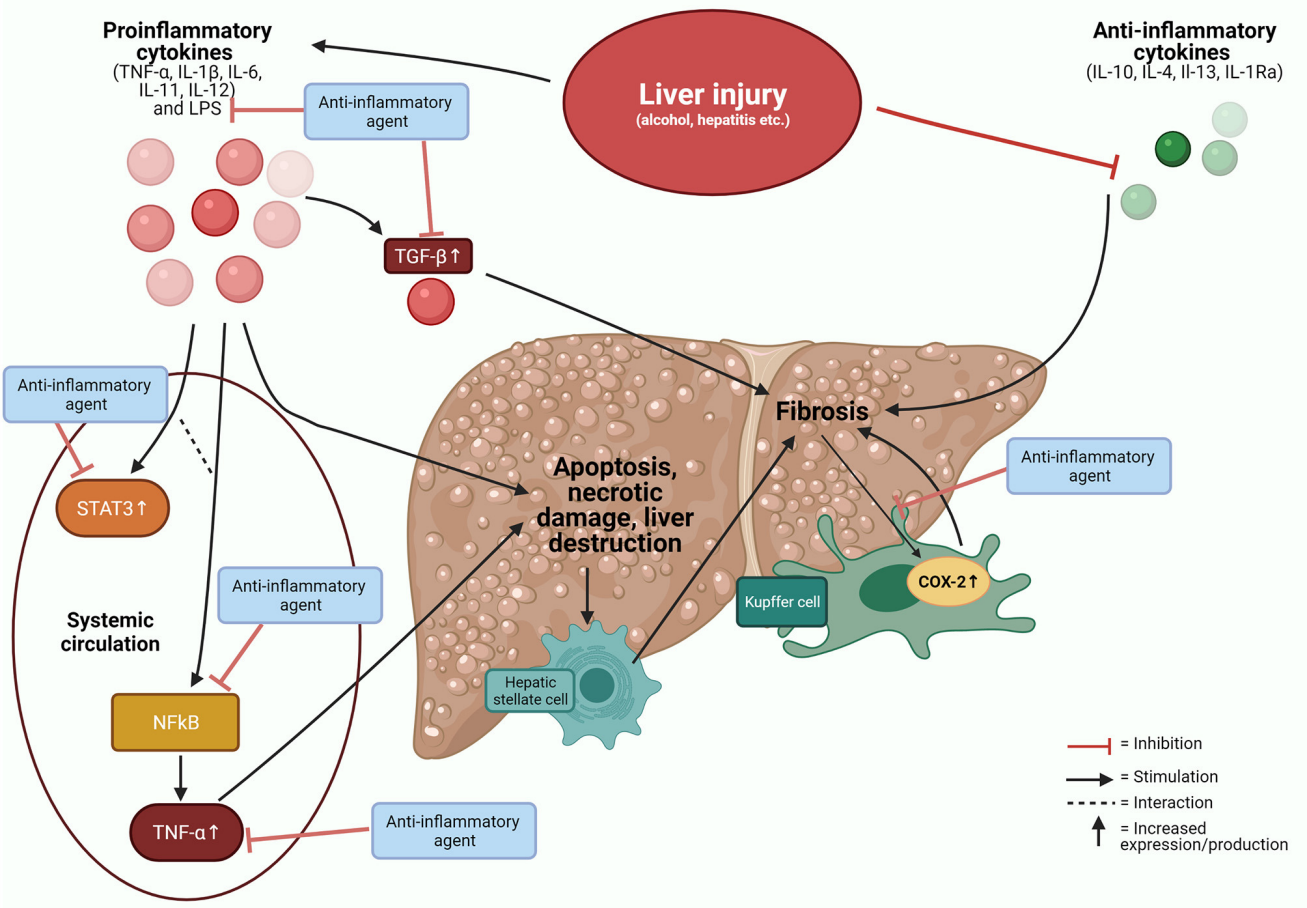
Cytokines in liver disease. Inflammatory responses on various liver injuries and potential targets for anti-inflammatory agents. COX-2, cyclooxygenase−2; IL, interleukin; LPS, lipopolysaccharide; NFkB, nuclear factor-kB; STAT3, signal transducer and activator 3; TGF, transforming growth factor; TNF, tumour necrosis factor.

Twelve different anti-inflammatory mediators acting on several pathways were evaluated in 30 animal studies and five human studies. Cytokines were most often used as a marker for inflammation. Cytokines are regulatory peptides released by activated cells and act as crucial mediators in immune and inflammatory disorders. Increasing evidence support a major role for several cytokines in liver diseases (Figure 2) (66).

The following agents are reported to interfere with the immune system, with potential beneficial effects:

*Celecoxib* (25–29, 67)*, aspirin* (30, 31)*, etanercept* (32)*, curcumin* (33–39)*, kahweol* (40, 41)*, pentoxifylline* (42, 68)*, diosmin* (42–44)*, glycyrrhizin arginine salt* (45)*, statins* (18, 19, 46–50)*, emricasan* (20–22, 51, 69) *and lanifibranor* (52) *and formayl receptor 2 agonist—WKYMVm* (53).

In addition, 27 human studies with various methodologies investigated the mechanisms but did not report anti-inflammatory endpoints. These are listed in Supplementary Table 2.

#### Celecoxib

Celecoxib is a cyclooxygenase-2 (COX-2) inhibitor used in the treatment of arthritis (70, 71). COX-2, an enzyme expressed due to inflammation, is increased in inflammatory, vascular endothelial lining, and expressed by Kupffer cells in the cirrhotic liver (72, 73). Celecoxib has anti-inflammatory effects to relieve cirrhosis complications and reduce portal hypertension in several rat studies (25–29, 67) (Supplementary Table 3a). Celecoxib was administered to prevent cirrhosis in experimental animal models where cirrhosis was induced by peritoneal injections of thioacetamide (TAA). However, no human studies with anti-inflammatory endpoints have been carried out with celecoxib.

#### Aspirin

Aspirin is a COX-inhibitor that acts on the nuclear factor kappa B (NFkB), which transcript adhesion molecules in endothelial cells and vascular smooth muscle cells, which affect macrophage and T lymphocyte adherence (30). In addition, aspirin may enhance interferon-α-induced growth inhibition and apoptosis in HCC (74), but few studies have investigated aspirin as a single treatment for cirrhosis.

In rats with TAA-induced cirrhosis, aspirin markedly reduces fibrogenesis with a macroscopic and histologic improvement of the liver tissue compared to controls (30, 31).

While biomarkers of inflammation were not assessed in these studies (30), serum bilirubin levels were significantly lower in the aspirin-treated cirrhotic rats than in the untreated cirrhotic rats (31).

So far, no human studies of aspirin as a treatment of inflammation have investigated specific molecular or anti-inflammatory biomarkers or inflammation cascades. Clinical endpoints investigated are listed in Supplementary Table 2 (75–77).

#### Etanercept

TNF-α is a cytokine produced in immune cells, and it is shown that hepatic signalling through the TNF-R1-receptor is essential for liver regeneration (78). TNF-α activates the NFkB-pathway, which mediates protective and anti-apoptotic effects but also initiates transcription of inflammatory mediator genes (79). However, TNF-α is also a proinflammatory mediator capable of inducing apoptosis and liver destruction (79). Etanercept is a TNF-α antagonist and an approved drug for autoimmune diseases, e.g., inflammatory bowel disease and rheumatoid arthritis. A single rat study (32) found that TNF-α levels were neutralised combined with a significantly lower expression of TNF-R1 by etanercept exposure. Surprisingly, a retrospective study of patients with various immune-related diseases receiving TNF-α-inhibitors found an increased hazard ratio for developing cirrhosis. Immunological pathways were not further assessed, and its endpoints are listed in Supplementary Table 2 (80).

#### Curcumin

Curcumin is a derivative of turmeric, which appears to have anti-inflammatory, antioxidant, plus anticarcinogenic effects (81).

Curcumin has an increasing effect on the signalling molecules nuclear factor erythroid 2-related factor 2 (Nrf-2), Nrf2-NFkB (measured as mRNA) and protein expression (34), and a decrease in the cytokines TGF-1β and TNF-α and in IL-10 (33). In addition, curcumin reduces alanine aminotransferase (ALT) levels in hamsters and rats with CCl_4_-induced cirrhosis (33, 35).

Curcumin may also reduce the expression of α-smooth muscle actin (α-SMA), a phenotypic marker of HSC-activation, and COX-2 combined with lactulose (35). In addition curcumin increases SIRT3, a sirtuin with a pivotal role in fatty acid oxidation and reduction of cellular reactive oxygen species (ROS) in the liver, which is decreased in cirrhotic rats (36). Furthermore, mRNA expression of the signalling molecules AMPK, CPT-1A, IDH2, and MnSOD was increased by curcumin, indicating reduced oxidative stress.

Curcumin was found to reduce protein expressions of eNOS, COX-2, VEGF, p-VEGFR2, and p-Erk in cirrhotic rats (37). The impact of curcumin on inflammatory markers was investigated by Cai et al. (38) and by Hernández-Aquino et al. (39) (Supplementary Table 3b). Curcumin appeared to decrease the levels of LPS-TLR4-related downstream inflammatory cytokines in the liver, specifically, TNF-α, IL-1β, IL-6, and CINC-1/IL-8. The decreases were mediated by decreased LPS levels and innate inflammation in the curcumin-treated group, not due to decreased LPS absorption but enhanced LPS clearance and detoxification in the liver (38). In addition, restoration of MMP-9, MMP-2, MMP-13m NFkB, IL-1, IL-10, TGF-β, connective tissue growth factor (CTGF), collagen, α-SMA, and Smad3+7 was also induced by curcumin as well as a decrement in activated hepatic stellate cells (39).

Overall, curcumin seems to decrease inflammatory responses, ameliorate fibrosis and portal hypertension, and attenuate splanchnic hyperdynamic circulation at least partly by inducing vasoconstriction through inhibition of eNOS and decreasing mesenteric angiogenesis via VEGF blockade.

In humans, a recent double-blind placebo-controlled trial demonstrated the effects of curcumin vs. placebo on disease severity in cirrhosis. However, specific anti-inflammatory effects have not been addressed in humans, see Supplementary Table 2 (82).

#### Kahweol

The LPS-induced inflammatory response is a crucial driver in systemic inflammation, most likely caused by bacterial translocation of PAMPs and DAMPs from the gut into blood circulation (6). The LPS signal transducer activates NFkB. Signal transducer and activator 3 (STAT3) is another transcriptional factor involved in the NFkB-pathway. Inhibition of these two factors could reduce the inflammatory responses (83).

Kahweol is a coffee-specific compound of coffee beans that exhibits anticarcinogenic, anti-tumour progressive, and anti-inflammatory properties (84), probably via affection of the NFkB and STAT3 signalling.

Seo et al. investigated kahweol's antifibrotic and anti-inflammatory effect on mouse liver Kupffer cells and hepatocytes *in vitro* (40). Kahweol was found to limit the production of IL-1α, IL-1β, IL-6, and TNF-α were reduced. Furthermore, this inhibitory effect was associated with the downregulation of LPS-stimulated phosphor-NFkB and STAT3.

Arauz et al. (41) investigated the effects of coffee in rats with TAA-induced cirrhosis. Coffee prevented a weight loss and limited the increase of ALT, alkaline phosphatase (ALP), and gamma-glutamyl transpeptidase observed in cirrhotic controls. In addition, significant amelioration of the cytokines TGF-β, CTGF, IL-10, and MMP-13 and α-SMA spikes evolved when coffee was administered to the rats. Thus, the action mechanisms are probably associated with antioxidant properties, mainly with coffee's ability to block the elevation of the profibrogenic cytokine (TGF-β) and the downstream effector CTGF.

No intervention studies in humans have yet explored the anti-inflammatory effects of coffee derivatives in patients with cirrhosis. However, one epidemiologic study is listed in Supplementary Table 2.

#### Pentoxifylline and Diosmin

Pentoxifylline is a non-selective phosphodiesterase inhibitor, which exhibits vasodilator activity on peripheral hepatic blood vessels (85). In addition, it exerts an anti-inflammatory regulation by affecting TGF-β- and tissue inhibitor metalloproteinase-1 (TIMP-1) expressions. Beneficial effects in humans with advanced liver disease have been described (68), and regulating effects of the hepatic stellate cell activity, is suggested to be related to the Hedgehog signalling pathway (86).

Diosmin is a natural flavone reported to prevent hepatic injury through inhibition of NFkB activation (42, 87). In bile duct ligated(BDL)-induced cirrhotic rats, pentoxifylline and diosmin have increased survival (42). A healing effect on the fibrotic markers HYP and TGF-β, and the oxidative markers malondialdehyde (MDA), SOD (superoxide dismutase), glutathione reductase (GSH), and nicotinamide adenine dinucleotide phosphate (NADPH) oxidase activity favoured both diosmin and pentoxifylline; see Supplementary Table 3c.

Diosmin has been investigated as a single treatment and in combination with sildenafil (43). Findings about its anti-inflammatory and antioxidant effects are described in Supplementary Table 3c. A rodent study (44) confirmed findings of regulation of TNF-α and NFkB activation in rats treated with ethanol and diosmin concomitant for 4 weeks. TNF-α was significantly elevated by ethanol and remitted by the concomitant addition of diosmin. NFkB was investigated by immunohistochemical staining, and the expression was markedly suppressed in diosmin-treated rat groups.

In a clinical trial, 329 patients with cirrhosis were randomised to treatment with pentoxifylline or placebo for 6 months (68). Pentoxifylline lowered complication rates of bacterial infections, renal insufficiency, hepatic encephalopathy, and gastrointestinal bleeding. Higher TNF-α baseline levels were associated with the development of complications in the absence of pentoxifylline.

#### Glycyrrhizin Arginine Salt

Glycyrrhizin is the primary active constituent of liquorice root. Liquorice has anti-inflammatory, spasmolytic, laxative, anti-depressive, anti-ulcer, and anti-diabetic effects (88). The statement is supported by a rodent study in which glycyrrhizin combined with arginine seems to protect against hyperammonaemia and hepatic encephalopathy (45).

The therapeutic effects of glycyrrhizin and arginine also change cytokine levels (TGF-β1 and TNF-α), antibodies against matrix metalloproteinases, and biochemical markers of liver function. In this study, significant changes were found to recommend glycyrrhizin arginine salt treatment. Results for its anti-inflammatory effects are listed in Supplementary Table 3d.

#### Statins

Statins have proved antioxidative, antiproliferative, and anti-inflammatory properties and a capacity to improve endothelial function and stimulate neoangiogenesis (89, 90). Statins decrease leukocyte adhesion to endothelial and epithelial cells by inhibiting expression and binding of the integrin LFA-1 and the intercellular adhesion molecule 1 (ICAM-1). Statins also decrease NFkB production, and hence the release of proinflammatory cytokines such as TNF-α and IL-6. This chain of action blocks critical proteins required to form lipid rafts and immune cell activation and growth. They also reduce the levels of oxidative stress (91).

Studies in rats with experimental-induced cirrhosis have shown that statins may prevent LPS-induced ACLF-derived complications and prolong survival. Moreover, statins increase the hepatic sinusoidal function, protect against endothelial dysfunction and the harmful effects of hypovolemic insults. Finally, statins normalise inflammatory markers during critical events such as ACLF and hypovolemia (46, 47) (see Supplementary Table 3e). A significant effect of simvastatin, when combined with bone-marrow-derived mesenchymal stem cells, has been demonstrated with amelioration of fibrosis (50) (see Supplementary Table 3e). Atorvastatin also reduces portal pressure in CCl_4_-cirrhotic rats (48). In human cirrhotic liver samples, Sonic hedgehog (Shh) and Glioma-associated oncogene family zinc finger-2 (Gli-2) mRNA levels, as well as protein expressions, increases (48). Atorvastatin treatment significantly downregulated the hedgehog components Shh and Gli-2 in the BDL and CCl_4_-cirrhotic models. Likewise, mRNA levels of α-SMA, collagen-1, and vimentin decreased after atorvastatin treatment.

In contrast, one rat study did not find a significant amelioration of cirrhosis on treatment with atorvastatin or rosuvastatin (49).

Simvastatin may increase the hepato-splanchnic output of nitric oxide products in patients with cirrhosis, thereby improving portal hypertension (18).

Randomised clinical trials have investigated beneficial effects on clinical outcomes such as liver function, rebleeding from oesophageal varices, and survival (92–94), and one ongoing multi-centre trial is also prospectively investigating the potential reduction of hepatic decompensation (19). However, no human studies have explored anti-inflammatory mechanisms in detail; hence, the therapeutic effects of statins are not yet completely understood [for clinical endpoints in human studies (90, 92–104) see Supplementary Table 2].

#### Emricasan

Emricasan is an oral pan-caspase inhibitor with alleviating impact on apoptosis, inflammation, and fibrosis in animal models of liver injury.

Studies have demonstrated how emricasan can inhibit hepatic cell death with reductions in caspase-3-activity in CCl_4_-cirrhotic rats, while reducing portal hypertension and hepatic microvascular dysfunction in rats with advanced cirrhosis is also described (51). In addition, three human studies have explored the clinical effects of emricasan (20–22).

Frenette et al. (20) administered emricasan 25 mg in a randomised, double-blinded, placebo-controlled trial. Emricasan reduced cleaved keratin-18 (a marker of apoptosis) relative to placebo, although insignificant, but caspase 3/7 and flCK-18 levels reduced significantly. Garcia-Tsao et al. (21) likewise found reductions in cleaved cytokeratin 18 and caspase-3/7 after 28 days of treatment with emricasan in 22 cirrhotic patients. Supplementary Table 3 lists anti-inflammatory marker results for the rodent studies.

A recent clinical trial concerning NASH-related cirrhosis by the same authors (22) showed the same biomarker patterns described above. However, the clinical effects on liver biochemistry and portal hypertension were not observed in patients with decompensated NASH-related cirrhosis (69) (see Supplementary Table 2).

#### Lanifibranor

Peroxisome proliferator-activated receptors (PPARs) are present in mammals in three isoforms, and all isoforms have a role in maintaining liver function (105). The pan-PPAR agonist lanifibranor has shown potential to alleviate models of mild liver injury and non-alcoholic fatty liver disease. We found one study of the effects of lanifibranor on cirrhotic rats and on cirrhotic human hepatic cells *in vitro*. Lanifibranor ameliorated fibrosis and portal hypertension in the rats in addition to significant anti-inflammatory effects (Supplementary Table 3f) and showed promising results in human hepatic cells. However, no human clinical trials were found.

#### Formyl Peptide Receptor 2 Agonist—(WKYMVm)

Hexapeptide WKYMVm (Trp-Lys-Tyr-Met-Val-D-Met) is a ligand of the formyl peptide receptor 2. It exhibits anti-inflammatory and angiogenic properties in multiple disease models. The WKYMVm peptide improves vascular remodelling and inhibits fibrosis in a rat model of hepatic failure (53) (See Supplementary Table 3g). Furthermore, WKYMVm enhances hepatic function by upregulating the expression of hepatic function markers. These data suggest that the WKYMVm peptide modulates liver function and vascular regeneration in rodent hepatic failure. No human trials are described.

### Antioxidants

Seventeen animal studies investigated the effects of five different antioxidant mediators, three of which were investigated in human studies with clinical endpoints): *Curcumin* (33–38)*, pentoxifylline* (42, 68), and *diosmin* (42–44) *are described above. Mitoquinone* (54, 55) and *silymarin* (56–60, 106) are described below.

**Figure 3:**
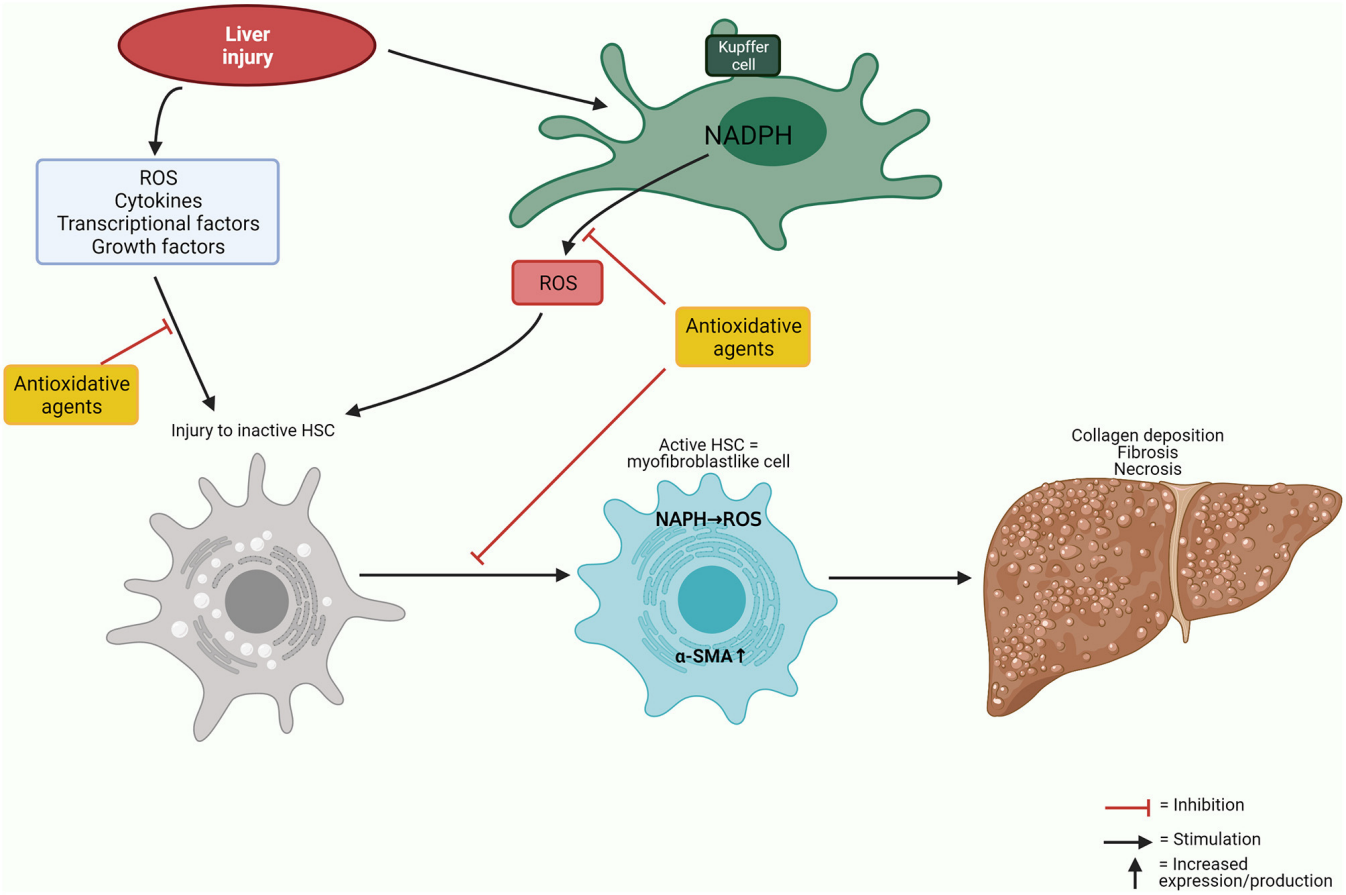
Antioxidative mechanisms. Simplified illustration of oxidative and antioxidative mechanisms affecting liver cells. α-SMA, α-smooth muscle actin; HSC, hepatic stellate cell; NADPH, nicotinamide adenine dinucleotide phosphate; ROS, reactive oxygen species.

Antioxidant agents seem to attenuate hepatic fibrosis in rodent models (107). The mechanism is partly due to the influence on the activation of hepatic stellate cells, which induce extracellular matrix deposition (Figure 3). Several agents have supposed antioxidant effects demonstrated by measurements of reactants as MDA, NADPH oxidase, Nrf2, Keap 1, NFkB, and IkB. NADPH oxidase is highly expressed by Kupffer cells and generates high amounts of ROS during early liver injury. In addition, hepatic stellate cells also seem to express NADPH—generating ROS, which mediates fibrogenic factors (107).

#### Mitoquinone

Mitochondrial dysfunction appears to play a crucial role in the development and progression of liver cirrhosis. Cirrhotic livers exhibit increased ROS produced by mitochondria. Mitoquinone is a mitochondria-targeted antioxidant, which might relieve the damaging effects of ROS within cirrhotic livers (54, 55). Two rat studies have assessed the effects of treatment with mitoquinone. Thus, Vilaseca et al. (54) found a relieving effect on portal hypertension in rats, as well as on fibrosis and oxidative markers.

Mitochondrial superoxide content was significantly higher in hepatic stellate cells and hepatocytes from cirrhotic rats but not in sinusoidal- or Kupffer cells compared to non-cirrhotic rats, and this effect appeared to be dose-dependent. Similar effects on HSC activity were found in human liver cells exposed to mitoquinone.

Mitoquinone also reduced oxidative stress and reduced portal pressure and intrahepatic vascular resistance in rats with CCl_4_-induced cirrhosis. Mitoquinone resulted in a significant reduction in hepatic fibrosis, which points to a potential clinical value of this drug. The activity of the hepatic stellate cells was assessed by the expression of profibrogenic genes and α-SMA, and both markers reduced significantly in cirrhotic rats.

Finally, inflammatory markers in *in vivo* models were measured, and iNOS, IL-6, and IL-1β were all reduced significantly.

In another study of BDL rats, Turkseven et al. (55) also investigated the effects of mitoquinone. Treatment with mitoquinone prevented inflammation, hepatocyte necrosis, and progression of fibrosis. Initially, bile-duct ligation of the rats led to increased gene expression (Qr-PCR) of inflammatory and oxidant markers, and these responses were reduced by mitoquinone. Collagen type col1α1, TGF-β, TNF-α, IL-6, IL-1β, and levels of circulatory TNF-α were all reduced. Furthermore, mitoquinone reduced the protein carbonylation, an indicator of irreversible oxidative protein modification, in cirrhotic rats. Mitoquinone normalised the gene expression of the mitochondrial antioxidant Mn-SOD, Cu/ZnSOD, and catalase impaired by cirrhosis.

Parkin protein expression in mitochondria is an indicator of the removal of dysfunctional mitochondria by autophagy. Parkin protein expression decreases in cirrhosis but increases in rats treated with mitoquinone (55).

Mitoquinone seems to possess both anti-inflammatory and antioxidant effects in human cells and rodent models, but no human studies have yet verified these effects.

#### Silymarin

Silymarin is an extract of the plant *Silybum marianum* (milk thistle), the main compound being silybin. Silymarin has a low bioavailability and lack solubility in water. Silybin acts by turning off proinflammatory signals derived from NFkB-activation (which is involved in the induction of TNF-α, IL-1, IL-6, and GM-CSF) and induces apoptosis. Silymarin's antioxidant activity is related to its free radical-scavenging and lipid peroxidation inhibition, as demonstrated *in vivo* and *in vitro* (108).

We identified five rodent studies that have assessed the effects of silymarin in cirrhosis models.

Ali et al. (58) investigated the modulatory effects of curcumin, silybin-phytosome, and alpha-R-lipoic acid in rats with TAA-induced cirrhosis. TAA was given at the same time as the intervention. Glutathione depletion, collagen deposition, matrix metalloproteinase-2 activity, TGF-β1 levels and heat shock protein-47 gene expressions- all factors believed to be involved in the development of cirrhosis-, were partially blocked by the combination therapy with curcumin, silybin-phytosome, and alpha-R-lipoic acid. Thus, therapy increased ROS generation and inhibited the activation of hepatic stellate cells, thereby preventing liver cirrhosis.

Zaidi et al. (56) also evaluated the effects of silymarin on rats with TAA-induced cirrhosis. Antioxidant activity was reduced as superoxide dismutase (SOD) and GSH were low, and MDA (measure of lipid peroxidation) and catalase were increased before treatment was initiated. Conversely, silymarin restored SOD and GSH, MDA, and catalase activity.

A possible synergistic effect between silymarin and lactulose has been investigated in a cirrhotic rat model (57) with no significant difference found between treatment groups according to their necro-inflammatory scores.

A combination of silymarin and the amino acid taurine was assessed in a study on CCl_4_-induced cirrhosis (59). Silymarin alone and silymarin with taurine restored the TBARS (thiobarbituric acid reactive substances) levels, and the combination treatment significantly reduced NO levels and NOS activity. However, activities of SOD, glutathione peroxidase (GPx), and glutathione reductase (GR) increased significantly in all treatment groups. In addition, glutathione-S-transferase (GST) and reduced glutathione (GSH) increased in rats treated with silymarin alone or the combination treatment.

The cytokines TNF-α, TGF-β1, IL-6, and the proteins leptin and resistin elevated in the cirrhotic model, while adiponectin reduced. All three treatments reduced TGF-β1, IL-6, and leptin, but only taurine and the combination taurine and silymarin reduced TNF-α and resistin.

Finally, a combination of bone-marrow-derived stromal cells and silymarin ameliorated liver tissue damage in a CCl_4_-cirrhotic rat model through immunoregulatory activities. However, antioxidative markers were not investigated in this study (60).

Only a single cohort study of silymarin has been carried out in humans. Fathalah et al. (106) investigated the effects of high-dose silymarin in decompensated chronic hepatitis C virus (HCV)-cirrhotic patients. The main results improved biochemical liver parameters and Child-Pugh score; however, no oxidative markers were investigated (see Supplementary Table 2).

### Gut Microflora and the LPS Pathway

Gut dysbiosis with translocation of bacteria and the bacterial product might play a role in the development of complications of cirrhosis. Alleviation of the dysbiosis in the gut flora of patients with cirrhosis, and the effects on LPS, supports that counteracting anti-inflammatory mechanisms are beneficial in cirrhosis.

Our search resulted in four animal studies, two human studies reporting effects on inflammatory markers, and two human studies where only clinical endpoints were considered. Thus, we found three different gut microbial modulation therapies, namely *Faecal microbiota transplantation* (23, 24, 62, 109)*, kahweol* (40, 41) *as described earlier, and artesunate* (63).

#### Faecal Microbiota Transplantation (FMT)

Among pertinent mechanisms, an increase in LPS leads to hepatocyte damage, which stimulates hepatic macrophages and increases the release of IL-1, IL-6, and TNF-α. Several pathways are involved in the promotion or counteraction of chronic inflammation (40). For example, kahweol affects the LPS pathway in the gut resulting in anti-inflammatory effects (described above). Liu et al. explored the effects faecal microbiota transplantation (FMT) from humans to germ-free and conventional mice (62). They found reduced neuroinflammation and microglial activation and dysbiosis 15 days after FMT exposure, whereas liver inflammation was unaffected. Higher degrees of neuroinflammation in mice regardless of their cirrhosis state was found with faecal microbial colonisation from humans with cirrhosis as compared with mice exposed to colonisation from healthy humans. Bajaj et al. investigated the safety of FMT capsules in patients with cirrhosis and recurrent HE in a Phase 1 randomised, placebo-controlled trial (23). FMT improved duodenal mucosal diversity, dysbiosis, and expression of duodenal antimicrobial peptide (AMP) and reduced lipopolysaccharide-binding protein (LBP). Subsequently, a trial was constructed based on the same cohorts elucidating the effects on inflammatory markers: IL-6 and LPS-binding protein, and bile acids in serum (24). Four weeks of FMT decreased levels of serum IL-6 and LBP compared to the placebo group. In the FMT group, greater deconjugation and secondary bile acid formation were found. In an ongoing study, Woodhouse et al. (109) currently assess if FMT in patients with advanced cirrhosis is effective, feasible, and safe.

#### Artesunate

Artesunate is an extract of the Chinese herb “Artemisia annua,” which has historically been used as an antimalarial drug. It is assumed to affect the pathological bacterial translocation (63), which is thought to be the key driver of spontaneous infection in patients with cirrhosis (110). For example, prophylactic antibacterial treatment is often indicated in patients with ascites and risk of spontaneous bacterial peritonitis. A single study assessed the effects of artesunate in rats with CCl_4_-induced cirrhosis (63). Thus, artesunate decreased IL-6 and TNF-α in the cirrhotic liver at week 4, 6, and 8, indicating an effect on inflammatory responses. Microbial diversity in the artesunate group, as compared to controls was increased at week 4 and reduced at weeks 6 and 8. Bacterial genomic DNA products reappeared in rats treated with artesunate after 4 weeks, unlike in the cirrhotic rats not treated with artesunate. No bacteria were detected in the blood in either group. Thus, artesunate decreased the occurrence of bacterial translocation significantly.

No human studies have yet been conducted, but the primary impression of the effects of artesunate is promising according to alleviating the inflammatory factors and the dysbiosis of gut microbiota in cirrhosis.

### Deactivation of Hepatic Stellate Cells

Several of the therapeutic agents described are hypothesised to have multiple impacts on different homeostatic and pathophysiological pathways. For example, enoxaparin (30, 31, 64, 65, 111) and tanshinone (61) may have effects that have not yet been classified as direct antifibrotic mechanisms and do not fit into a concrete anti-inflammatory or antioxidative mode of action.

#### Enoxaparin

Enoxaparin has both anticoagulant and antithrombotic effects (112). A rat experimental study observed a reduction of proliferation and activation of hepatic stellate cells (30). In addition, it has been demonstrated that patients with cirrhosis more frequently exhibit a prothrombotic state than a hypocoagulative state (113, 114).

Enoxaparin has been proven to reduce portal pressure in cirrhotic rats, implying effects beyond the anti-thrombotic (65). Short-term treatment of cirrhotic rats with enoxaparin showed a significant reduction of superoxide content, α-SMA, and mRNA of pro-collagen I and liver fibrosis. In addition, the oxidative stress levels were lower, and fibrosis reduced by 25% after enoxaparin treatment.

However, Fortea et al. (64) did not find an amelioration of fibrosis, biochemical parameters, hepatic endothelial dysfunction, or portal hypertension after enoxaparin treatment of cirrhotic rats. On the contrary, the therapeutic dose of enoxaparin did decrease survival in rats with CCl_4_-induced cirrhosis. Enoxaparin as a preventive therapy for portal venous thrombosis in patients with Child-Pugh B-C cirrhosis has been suggested by the authors of one study (111), favouring enoxaparin compared to no treatment. It was found safe and preventive for thrombosis for 34 patients treated for 48 weeks. In addition, the frequency of decompensation reduced, and survival increased in the enoxaparin group.

#### Tanshinone

Salvia miltiorrhiza (S. miltiorrhiza) is a Chinese herb comprising multiple compounds. Tanshinone is extracted from S. miltiorrhiza and is described as a natural antioxidant with hepatoprotective, antifibrotic, and anticancerogenic effects. In addition, it is supposed to induce stem cell proliferation and differentiation (115). A single rat study investigated the effects in a cirrhotic model (61), where tanshinone improved the histological injury, serological tests, and increased expression of markers indicating newly proliferated stem cells. These effects appeared to be caused by promoting proliferation and differentiation of endogenous liver stem cells. No human trials were found.

## Discussion

In the present review, we have identified and explored possible anti-inflammatory and antioxidant agents as potential drug candidates to interfere with the fibrogenesis processes and thereby alleviate the development and perpetuation of complications of cirrhosis. In cirrhotic rodent models we have found promising indices of beneficial anti-inflammatory and antioxidative effects of the COX-2 inhibitor celecoxib, aspirin, curcumin, kahweol, pentoxifylline, diosmin, statins, emricasan, and silymarin. Few indices of effects of etanercept, glycyrrhizin arginine salt, and mitoquinone were found. In addition, FMT is a growing field with the potential to alleviate cirrhosis by a beneficial regulation of the gut flora.

The main limitation of the present review is the lack of human studies assessing anti-inflammatory agents in cirrhosis. The lack makes it difficult to assess the clinical efficacy of the agents discussed and to compare the effects between different studies. The literature offers many experimental studies in rodents where multiple beneficial effects on cirrhosis regarding fibrosis are reported (see Supplementary Table 3). However, extrapolating experimental animal studies into a clinically relevant setting is problematic. Nevertheless, several different agents were identified, and many of these showed potential curing or relieving mechanisms and effects in the applied models. However, most of the rodent studies are not comparable since especially the method of inducing cirrhosis differs. The procedures, the duration, and the timing of adding the experimental agents differ vividly.

There are important conflicting results for enoxaparin, statins, and etanercept. In particular, one human study investigating etanercept found significant harmful effects (80). One rat study of statins (49) and enoxaparin (64) showed no intervention effects. Curcumin seems promising in both rodent and human clinical trials. Anti-inflammatory and antifibrotic effects, as well as a positive impact on portal pressure and hemodynamic in rodents combined with favourable clinical outcome in cirrhotic patients, indicates a convincing potential (33–38, 82). Curcumin also has potential effects on quality of life (116). Silymarin is also studied in a different setting and shows potential anti-inflammatory and antioxidative effects in rodents and clinical improvement in patients. Emricasan showed promising alleviating effects in both rodent and human studies on several parameters. Promising clinical effects (20–22) are shown, but only caspase and flCK-18 are measured as anti-inflammatory markers. Hence, the mechanisms are still largely unexplored, and future studies with inflammatory and clinical endpoints are warranted.

Several retrospective studies have suggested that statins can improve mortality in cirrhosis (90, 95, 103). Clinical studies of the anti-portal hypertensive effects of statins have provided encouraging results in recently published trials (93, 97, 102, 117), while other studies have reported a reduced risk of decompensation and death (89, 90, 92, 94), and that statins might reduce the risk of infections in patients with cirrhosis (96). One study also described improved survival during infections among cirrhotic patients undergoing continuing treatment with statins (104). Human studies exploring anti-inflammatory mechanisms in cirrhosis should be highly encouraged.

The anti-inflammatory effects of statins seem to be steps ahead of other agents concerning testing in human trials, mainly due to the similar effect of statins on portal hypertension (93, 97, 102, 117). Novel research implies the anti-inflammatory effects of statins as key drives in lowering the portal pressure, but the immunological impact needs further exploration in humans (89, 91).

Dysbiosis in the gut flora is suspected as a precipitating factor in cirrhotic patients with infections who need antibiotic treatment. FMT could prove to be a valuable “post-treatment” after antibiotic exposure, restoring the potentially harmful effect antibiotics have on microbial diversity and function (118). Initial studies have focused on safety and organ function outcomes in cirrhosis (23), relevant in a complex disease entity. Studies on FMT without prior antibiotics are needed to assess the impact of gut dysbiosis and the inhibition or alleviation of inflammation in decompensated cirrhosis. Future studies are awaited (109) in the search for preventive and treating agents in cirrhosis.

Very few side effects are reported for the main part of the described agents, and safety deserves a primary focus in future investigations of anti-inflammatory agents. Only two safety studies regarding celecoxib are conducted (119, 120).

Real-life clinical trials exploring anti-inflammatory interventions and their safety in patients with liver cirrhosis are notably missing in the literature. Furthermore, data on the combination of anti-inflammatory or antioxidative markers and clinical outcomes are scarce. Human randomised clinical trials, preferably placebo-controlled, are the next step toward clinical application of anti-inflammatory agents. As most inflammation markers are easy to sample by blood tests, this should be possible and feasible to add to clinical protocols.

Autologous macrophage therapy is another promising treatment that seems safe (121); the effects of which are yet to be fully investigated. This is also the case for other autologous cell transplantation, such as mesenchymal stromal cell therapy (122).

In conclusion, we recommend further study of the inflammatory, oxidative, microbiological, and immunological mechanisms and pathways responsible for disease progression in cirrhosis. Known and novel compounds with potential healing effects in cirrhosis are identified and require further exploration. In general, the literature encloses very few human clinical trials on the aspects, and the need for studies is growing. Future studies should include anti-inflammatory biomarkers and clinical endpoints in combination to assess potential immunological agents in the treatment of cirrhosis.

## Data Availability Statement

The original contributions presented in the study are included in the article/Supplementary Material, further inquiries can be directed to the corresponding author/s.

## Author Contributions

TK, NK, and SM contributed to conception and design of the review. TK performed the literature searches and wrote the first draught of the manuscript. NK and TK assessed the literature searches. All authors critically revised the litterature and contributed to manuscript revision, read, and approved the submitted version.

## Conflict of Interest

The authors declare that the research was conducted in the absence of any commercial or financial relationships that could be construed as a potential conflict of interest.

## Publisher's Note

All claims expressed in this article are solely those of the authors and do not necessarily represent those of their affiliated organizations, or those of the publisher, the editors and the reviewers. Any product that may be evaluated in this article, or claim that may be made by its manufacturer, is not guaranteed or endorsed by the publisher.

## Abbreviations

ACLF: acute on chronic liver failure
α-SMA: α-smooth muscle actin
ALT: alanine aminotransferase
ALP: alkaline phosphatase
AMP: antimicrobial peptide
AST: aspartate aminotransferase
BDL: bile duct ligation
CCl_4_: carbon tetrachloride
cCK18: cleaved cytokeratine-18
COX: cyclooxygenase
CTGF: connective tissue growth factor
eNOS: endothelial nitric oxide synthase
flCK18: full-length cytokeratine 18
FMT: faecal microbiota transplantation
GPx: glutathione peroxidase
GR: glutathione reductase
GSH: glutathione reductase/reduced glutathione
GST: glutathione-S-transferase
HBV: Hepatitis B virus
HCC: hepatocellular carcinoma
HCV: Hepatitis C virus
HE: hepatic encephalopathy
HRS: hepatorenal syndrome
HSC: hepatic stellate cells
HVPG: hepatic venous pressure gradient
HYP: hydroxyproline
ICAM-1: intercellular adhesion molecule 1
IL-6: interleukine-6
Keap-1: Kelch-like ECH-associated protein 1
KLF2: Krüppel-like factor 2
LDL: low density lipoprotein
LFA-1: lymphocyte function-associated antigen 1
LPS: lipopolysaccharide
MDA: malondialdehyde, measure of lipid peroxidation
MSCs: mesenchymal stem cells
NADPH: nicotinamide adenine dinucleotide phosphate
NFkB: nuclear factor-kB
NO: nitrogen oxide
NOS: nitrogen oxygen synthase
Nrf2: Nuclear factor erythroid 2-realted factor 2
PARs: protease activated receptors
PDGF: platelet derived growth factor
PPARα: peroxisome proliferator-activated receptor-α
RCT: randomised controlled trial
ROS: reactive oxygen species
SBP: spontaneous bacterial peritonitis
SOD: superoxide dismutase
STAT3: signal transducer and activator 3
Supp.: supplementary
TAA: thioacetamide
TBARS: thiobarbituric acid reactive substances
TGF-β: transforming growth factor-β
TNF-α: tumour necrosis factor-α
VEGF: vascular endothelial growth factor
WKYMVm: a hexapeptide.
