# Supplementary material for: Novel Anti-inflammatory Treatments in Cirrhosis. A Literature-Based Study
Source: Front Med (Lausanne). 2021 Sep 23;8:718896. doi: 10.3389/fmed.2021.718896 (PMC8495012; doi:10.3389/fmed.2021.718896)
Supplement: Supplementary file 1 [file Table_1.DOCX]

**Supplementary Table 1:** Search strategy and results

| **Initial broad searches, updated 16^th^ of May 2021** | | |
| --- | --- | --- |
| **Search** | **Hits** | **Assessed** |
| ’chronic liver disease’ ’anti-inflammatory’ ‘drugs’ | 972 | 58 |
| ‘cirrhosis’ ‘inflammation’ | 42,596 | - |
| ‘liver cirrhosis’ ‘inflammation’ | 9,068 | 418 |
| ‘liver cirrhosis’ ‘anti-inflammatory’ | 3,742 | 172 |
| ‘liver cirrhosis’ ‘cure’ | 1,062 | 666 |
| PubMed Search with MESH-terms;  ("liver cirrhosis"[MeSH Terms] OR ("liver"[All Fields] AND "cirrhosis"[All Fields]) OR "liver cirrhosis"[All Fields] OR ("hepatic"[All Fields] AND "cirrhosis"[All Fields]) OR "hepatic cirrhosis"[All Fields]) AND ("anti-inflammatory agents"[Pharmacological Action] OR "anti-inflammatory agents"[MeSH Terms] OR ("anti-inflammatory"[All Fields] AND "agents"[All Fields]) OR "anti-inflammatory agents"[All Fields] OR "antiinflammatory"[All Fields]) AND ("2015/04/25"[PDat] : "2020/04/22"[PDat]) | 413 | 45 |
| **Total** | **57,853** | **1,359** |

| **Initial searches lead to following specific drug searches; # indicates search of the drug AND ‘liver cirrhosis’ (13/11-2020-16/5-2021)** | | | | |
| --- | --- | --- | --- | --- |
| **Search** | **Hits** | **Assessed after primary filtration** | **Relevant from search of reference lists and bibliographies** | **Relevant** |
| #silymarin | 294 | 24 | - | 6 |
| #aspirin | 147 | 144 | 8 | 5 |
| #enoxaparin | 56 | 5 | 5 | 3 new + 2 from aspirin search |
| #fraxetin | 11 | 3 | - | 0 |
| #kahweol | 8 | 4 | - | 3 |
| #etanercept | 30 | 30 | - | 2 |
| #curcumin | 121 | 22 | - | 8 |
| #faecal microbiota transplantation | 72 | 8 | - | 4 |
| #artesunate | 8 | 8 | - | 1 |
| #celecoxib | 29 | 29 | 2 | 8 |
| #mitoquinone | 2 | 2 | - | 2 |
| #glycyrrhizin arginine salt | 1 | 1 | - | 1 |
| #pentoxifylline | 142 | 4 | 1 | 2 |
| #diosmin | 7 | 4 | - | 2 + 1 from pentoxifylline search |
| #statin | 344 | 43 | - | 21 |
| #emricasan | 17 | 7 | - | 5 |
| #baihe wuyao decoction | 1 | 1 | - | 0 |
| #substance p | 26 | 7 |  | 0 |
| #codonopis buleynana | 1 | 1 |  | 0 |
| #lanifibranor | 10 | 1 |  | 1 |
| #forsythoside B | 1 | 1 |  | 0 |
| #formyl peptide receptor 2 | 1 | 1 |  | 1 |
| #tanshinone | 8 | 1 |  | 1 |
| **Total** | **1,337** | **351** | **16** | **76** |
