# Supplementary material for: Novel Anti-inflammatory Treatments in Cirrhosis. A Literature-Based Study
Source: Front Med (Lausanne). 2021 Sep 23;8:718896. doi: 10.3389/fmed.2021.718896 (PMC8495012; doi:10.3389/fmed.2021.718896)
Supplement: Supplementary file 2 [file Table_2.docx]

**Supplementary Table 2:** Excluded human studies; Humans studies of anti-inflammatory drugs exploring clinical outcomes in cirrhosis (n=27)

| **Study ID** | **Species** | **Intervention** | **Methods** | **Aim** | **Results** |
| --- | --- | --- | --- | --- | --- |
| Jiang et al. 2016 | Human | Reported use of Aspirin in any amount in the past month | Cross-sectional analysis of preregistered data on 1,865 patients with chronic liver disease | To examine the association between aspirin and liver fibrosis | Aspirin was associated with sig. lower indices of fibrosis in adults with suspected chronic liver disease |
| Shin et al. 2019 | Humans | Aspirin 100 mg/day | Retrospective analysis from 949 patients with alcoholic cirrhosis | To investigate whether aspirin use lowers risk of HCC in alcoholic cirrhosis | Aspirin was associated with lower risk of HCC |
| Lee et al. 2019 | Humans | Aspirin (daily for at least 90 days, doses not specified) | Cohort study of 204,507 patients with chronic HBV | To investigate association of aspirin with HBV-related HCC risk. | Aspirin therapy may be associated with a reduced risk of HBV-related HCC |
| Walton et al. 2013 | Humans | Kahweol (questionnaire on average daily coffee consumption) | Cohort study of 286 patients from the liver outpatient department | Compare coffee consumption in patients with liver disease compared with orthopaedic outpatients and medical students. | Cirrhotic patients drank significantly less coffee than patients without cirrhosis. No correlation between ChildPugh and MELD scores with coffee consumption. |
| Lebrec et al. 2010 | Humans | Pentoxifylline (400 mg, three times daily for six months) | Randomized, placebo-controlled, double-blind trial of 335 patients with cirrhosis | Effects of pentoxifylline on complications and survival | Pentoxifylline reduced mortality in 2 months observation. No difference after 6 months. Increased probability of survival without complications when treated with pentoxifylline. |
| Huang et al. 2016 | Humans | Statins (all sorts of statins, exposure defined as 28 cumulative defined daily dose) | Population-based cohort study of 298,761 patients | Statin effect on survival and HCC incidence in patients with chronic HBV | Statin dose-dependent reduction in the risk of cirrhosis and its decompensation; IR: 0.561 vs. 1.338 and 0.190 vs. 0.411 pr. 100 person years |
| Motzkus-Feagans et al. 2013 | Humans | Statins (all sorts of statins, doses not specified) | American veteran cohort study of 19,379 patients | To estimate if statin prolongs time to infection among patients with cirrhosis. | Statins reduced infection rate |
| Bishnu et al. 2017 | Humans | Statins (20 mg atorvastatin for 30 days) | Open-label proof of concept study, 23 patients randomised | Propranolol and atorvastatin vs. propranolol alone to investigate change in portal pressure gradient | Significant decrease in hepatic venous pressure gradient when treated with combination |
| Kumar et al. 2014 | Humans | Statins (all sorts of statins, doses not specified) | Retrospective cohort of 243 patients | Mortality in cirrhotic patients | Statin was not associated with increased mortality, and may delay decompensation |
| Simon et al. 2016 | Humans | Statins (simvastatin, lovastatin, Fluvastatin, pravastatin, atorvastatin or cerivastatin, exposure defined as cumulative defined daily dose) | Retrospective cohort of 9,135 patients | Impact of statin on fibrosis progression and HCC in patients with HCV | Statin use was associated with a dose-dependent reduction in incident cirrhosis and HCC |
| Abraldes et al. 2009 | Humans | Simvastatin (20 mg for 14 days, increased to 40 mg thereafter for 14 days) | Randomized, double-blinded, controlled, clinical trial of 59 patients with cirrhosis and portal hypertension | Effects of simvastatin administration on HVPG and safety | Significant decrease of HVPG. Improvement in liver perfusion and function. |
| Abraldes et al. 2016 | Humans | Simvastatin (20 mg for 14 days, increased to 40 mg thereafter for up to 24 months) | Multi-center, double-blind, parallel clinical trial of 52 patients with cirrhosis and variceal bleeding. | Effects of Simvastatin on rebleeding rates and mortality. | Survival benefit for ChildPugh A and B groups when exposed to simvastatin. |
| Mohanty et al. 2015 | Humans | Statins (simvastatin, lovastatin, rosuvastatin, atorvastatin, pravastatin and Fluvastatin, doses according to prescriptions) | Retrospective cohort of 40,512 patients with HCV compensated cirrhosis | Effects of statins on decompensation and survival | 40% decreased risk of decompensation and death when taking statins |
| Chang et al. 2017 | Humans | Statins (all sorts of statins, exposure defined as 28 cumulative defined daily dose) | Retrospective cohort study of 1,350 patients with HBV-, HCV- and alcohol-related cirrhosis | Effect of statin on decompensation, mortality and HCC | Decrease the risk of decompensation, mortality and HCC by statin intake in a dose-dependent manner. |
| Elwan et al.  2018 | Humans | Statins 20-40 mg (simvastatin 20 mg for two weeks followed by 40 mg for two weeks) | Randomized controlled trial of 40 patients with cirrhosis in patients with cirrhosis prior and after simvastatin | Portal hypertension by Doppler ultrasound | Sig. reduction of portal hypertension index by sort term statin therapy (30 days) |
| Bang et al. 2017 | Humans | Statins (simvastatin, atorvastatin, rosuvastatin or combinations, retrospective claimed daily doses pr. day) | Register-based case-cohort of 5,417 patients with cirrhosis | Use of statins and mortality rate | Reduced risk of decompensation and death of cirrhosis when regular use of statins. |
| Pollo-Flores et al. 2015 | Humans | Statins (simvastatin 40 mg/day for three months) | Randomized, triple-blind, placebo-controlled trial of 24 patients with cirrhosis | Effects of simvastatin on HVPG and azygos vein blood flow | Clinically relevant decrease in HVPG when exposed to simvastatin. |
| Kaplan et al. 2019 | Humans | Statins (all sorts of statins– normalised to simvastatin-equivalent, exposure considered continuous if ≤15 days from the end of an individual dispensed supply to the start of the next) | Retrospective cohort study of 72,944 patients with cirrhosis | Effects of hyperlipidemia and statin exposure on mortality, hepatic decompensation and HCC development | Statin exposure was associated with an independent decrease of mortality (8-8,7%). Statin-naïve individuals with increase in total cholesterol had a 3,6% decrease in mortality. |
| Hung et al. 2019 | Humans | Statins (atorvastatin, rosuvastatin, fluvastatin, simvastatin, pravastatin, lovastatin and pitavasatin, doses not specified) | Population based cohort of 298,761 patients with cirrhosis | Effects of statins during infections | Statin use improved survival after infections |
| Fathalah et al. 2017 | Humans | Silymarin (420 or 1,050 mg for 12 weeks | Randomized, double-blind controlled trial of 62 patients with HCV decompensated cirrhosis | Effects of silymarin on clinical and biochemical status | Improvement of AST, ALT, bilirubin, albumin and INR levels – improved ChildPugh score in high dose regimen of Silymarin. |
| Woodhouse et al. 2018 | Humans | FMT (gastroscopic administration, one transplantation) | Randomized placebo-controlled trial of 24 patients with advanced cirrhosis. | Safety of faecal microbiota transplantation and effect on restoration of gut microbiome. | *No results yet.* |
| Villa et al. 2012 | Humans | Enoxaparin (4,000 IU/day for 48 weeks) | Randomized, open-label, controlled trial of 70 patients with cirrhosis | Safety and efficacy of enoxaparin in preventing portal vein thrombosis in patients with advanced cirrhosis. | Prevention of portal vein thrombosis, delay of decompensation and improved survival. |
| Nouri-Vaskeh et al. 2020 | Humans | Curcumin (1,000 mg/day for three months) | Randomized, double-blind, placebo-controlled trial, 70 patients with liver cirrhosis | Effects on MELD and Child-Pugh | Decrease in disease activity scores |
| Tang et al. 2020 | Humans | Etanercept (according to prescriptions; gaps of 84 days or fewer between use were considered continuous use) | Retrospective cohort study of 226,555 people from US database. | Evaluating impact of treatment with anti-TNF-α agents on developing new-onset cirrhosis and NAFLD and/or NASH | No beneficial effect of anti-TNF-α use for development of cirrhosis, NAFLD or NASH in patients with immune-related diseases. |
| Clària et al. 2005 | Human | Celecoxib (200 mg x 2)/Naproxen (500 mg x 2)/ placebo; every 12 hours, five doses in total | Double-blind, randomized, placebo-controlled trial of 28 patients with naproxen and placebo in patients with cirrhosis and ascites. | To compare effects of the COX-2 inhibitor celecoxib | Short-term administration of celecoxib does not affect decompensated cirrhosis. |
| Guevara et al. 2004 | Human | Celecoxib 200 mg, for four days | Pilot study of 9 patients with cirrhosis and ascites. | Effect of celecoxib on renal function in cirrhotic patients with ascites. | Short-term administration of celecoxib had no effect on renal function |
| Frenette et al. 2020 | Human | Emricasan 5 or 25 mg for 48 weeks | Double-blind, placebo-controlled study of 217 patients with decompensated NASH-cirrhosis | Effects on mortality, decompensation and MELD-Na score | Safe, but no effect on MELD-Na, INR, bilirubin, albumin or ChildPugh. |
