# Supplementary material for: Novel Anti-inflammatory Treatments in Cirrhosis. A Literature-Based Study
Source: Front Med (Lausanne). 2021 Sep 23;8:718896. doi: 10.3389/fmed.2021.718896 (PMC8495012; doi:10.3389/fmed.2021.718896)
Supplement: Supplementary file 3 [file Table_3.docx]

**Supplementary Table 3:** Inflammatory modulation by drugs in animal studies

Effects of drugs on inflammatory markers compared to induced cirrhosis in rodent models. *=significant effect according to authors.

| 1. **Celecoxib** | | | | | | | | | | | | | |  |  |  |  |  |  |  |  |  |  |  |  |  |  |  |  |  |  |  |  |
| --- | --- | --- | --- | --- | --- | --- | --- | --- | --- | --- | --- | --- | --- | --- | --- | --- | --- | --- | --- | --- | --- | --- | --- | --- | --- | --- | --- | --- | --- | --- | --- | --- | --- |
| **Rat treatment/Marker** | **Cytokines** | **COX-2** | | | **VEGF** | | | **pErk** | | | **Collagen** | | | | | **α-SMA** | | | | | | **Other** | | |  |  |  |  |  |  |  |  |  |
| Celecoxib, TAA.induced cirrhosis  (Gao et al. 2016) | TNF-α↓* and IL-6↓* in liver tissue | mRNA and protein ↓* | | |  | | |  | | | ↓* | | | | | ↓* | | | | | | LPS↓*, effect on intestinal tissue, T-cellsubset sig. raised in portal vein, intestinal permeability↓* | | |  |  |  |  |  |  |  |  |  |
| Celecoxib, TAA-induced cirrhosis  (Gao et al. 2013) | NA | ↓* | | | ↓* | | | ↓* | | | ↓* | | | | | mRNA of SMA↓* | | | | | | Decrease in portal pressure,  CD31-expression ↓*, Prostaglandin E2 ↓*, HIF-1α and c-fos↓* | | |  |  |  |  |  |  |  |  |  |
| Celecoxib,TAA-induced cirrhosis  (Wen et al. 2014) | TNF-α↓, IL-6↓, TGF-β1↓ | ↓* | | | NA | | | NA | | | NA | | | | | ↓* | | | | | | PGE2↓*, MMP-2↓*, MMP-9↓*, Phospho-Smad2/3↓*, Snail1↓*, FSP-1↓*, vimentin↓*, restoring the levels of E-cadherin* | | |  |  |  |  |  |  |  |  |  |
| Celecoxib and octreotide, TAA-induced cirrhosis  (Gao et al. 2016) | NA | ↓* | | | ↓* | | | ↓* | | | ↓* | | | | | mRNA of SMA↓* | | | | | | Decrease in portal pressure, CD31-expression↓*, Prostaglandin E2↓*, HIF-1α and c-fos↓* | | |  |  |  |  |  |  |  |  |  |
| Celecoxib, TAA-induced cirrhosis  (Su et al. 2020) | NA | ↓* | | | NA | | | ↓* | | | NA | | | | | NA | | | | | | Caspase3↓*, Caspase 12↓*, GRP78↓*, CHOP↓*, Hyp↓*. UPR related pathway proteins: XBP1↓*, ATF4↓*, IRE1↓*, ATF6↓* | | |  |  |  |  |  |  |  |  |  |
| Celecoxib, TAA-induced cirrhosis  (Tang et al. 2021) | TNF-α↓*, IL-1β↓*, IL-6↓*, TGF-β1↓* | ↓* | | | NA | | | NA | | | NA | | | | | NA | | | | | | Sig. decrease of extracellular matrix, decrease of spleen weight/body weight. NOX-4↓, non-sign. decrease of IgM and IgG | | |  |  |  |  |  |  |  |  |  |
| 1. **Curcumin** | | |  |  |  |  |  |  |  |  |  |  |  |  |  |  |  |  |  |  |  |  |  |  |  |  |  |  |  |  |  |  |  |
| **Rodent treatment/Marker** | **Cytokines** | **Nrf-2 mRNA** | | **NF-kB-p65** | | | | | | **iNos** | | | **eNos** | | | | **COX-1** | | **COX-2** | **VEGF** | | | | | | **pErk** | | **Phospho-Akt** | | **Other** | |  |  |
| Curcumin to BDL rats (Hsu et al. 2018) | NA | NA | | NA | | | | | | ↓ | | | ↓* | | | | ↓ | | ↓* | ↓* | | | | | | ↓* | | ↑ | |  | |  |  |
| Curcumin to CCl_4_ rats, (Cai et al. 2017) | TNF-α↓*, IL-1β↓*, IL-6↓*, CINC-1/IL-8↓* (in serum + liver tissue) | NA | | NA | | | | | | NA | | | NA | | | | NA | | NA | NA | | | | | | NA | | NA | |  | |  |  |
| Curcumin, doxazosin and/or carvedilol to CCl_4_ cirrhotic hamsters  (Macías-Pérez et al. 2019) | NA | Pos. cells to Nrf-2↑* (for Cu+Ca+D and Cu+D, and Cu alone) | | Pos. cells to NF-kB↓* (for Cu+Ca) | | | | | | NA | | | NA | | | | NA | | NA | NA | | | | | | NA | | NA | |  | |  |  |
| Curcumin to CCl_4_ rats,  (Hernandez-Aquino et al. 2020) | IL-1β↓*, IL-10↓*, TGF-β↓* | NA | | ↓* | | | | | | NA | | | NA | | | | NA | | NA | NA | | | | | | NA | | NA | | CTGF ↓*MMP-9↓*, MMP-2↓*, Col-1↓*, MMP-13↓*, Smad-7↓*, α-SMA↓* and Smad-3↓*. | |  |  |
| 1. **Pentoxifylline and Diosmin** | | |  |  |  |  |  |  |  |  |  |  |  |  |  |  |  |  |  |  |  |  |  |  |  |  |  |  |  |  |  |  |  |
| **Rat treatment/Marker** | **Cytokines** | **MDA** | **SOD** | | | | **GSH** | | **NADPH-oxidase** | | | **Cytoglobin** | | | | **Nrf-2 mRNA** | | **Keap-1** | | | **P38-MAPK** | | **NF-kB-p65** | **iNos** | **eNos** | | **Other** | | | | | |  |
| Diosmin  (Ali et al. 2018) |  | ↓* | ↑* | | | | ↑* | | ↓* | | | ↑* | | | | ↑* | | ↓* | | | ↓* | | ↓* | ↓* | ↑* | |  | | | | | |  |
| Pentoxifylline  (Ali et al. 2018) |  | ↓* | ↑* | | | | ↑* | | ↓* | | | ↑* | | | | ↑* | | ↓* | | | ↓* | | ↓* | ↓* | ↑* | |  | | | | | |  |
| Pentoxifylline + Diosmin  (Ali et al. 2018) |  | ↓* | ↑* | | | | ↑* | | ↓* | | | ↑* | | | | ↑* | | ↓* | | | ↓* | | ↓* | ↓* | ↑* | |  | | | | | |  |
| Diosmin (high and low dose)  (Tahir et al. 2012) | TNF-α↓* (both doses) | ↓* (both doses) | NA | | | | ↑*  (both doses) | | NA | | | NA | | | | NA | | NA | | | NA | | ↓* (both doses) | ↓* (both doses) | NA | | Alcohol dehydrogenase↓* (high dose), COX-2↓* (both doses), CYP 450 2E1↓* (both doses), Xanthine oxidase↓* (both doses), GPx↑*(both doses), GR↑*  (both doses), Catalase↑*  (both doses) | | | | | |  |
| Diosmin  (Ali et al. 2018) | TGF-β ↓* | ↓* | ↑* | | | | ↑* | | NA | | | ↑* | | | | ↑* | | ↓* | | | ↓ | | ↓ | ↓ | ↑ | | HYP↓* | | | | | |  |
| Sildenafil  (Ali et al. 2018) | TGF-β ↓* | ↓* | ↑* | | | | ↑* | | NA | | | ↑* | | | | ↑ | | ↓* | | | ↓ | | ↓ | ↓ | ↑ | | HYP↓* | | | | | |  |
| Sildenafil + Diosmin  (Ali et al. 2018) | TGF-β ↓* | ↓* | ↑* | | | | ↑* | | NA | | | ↑** | | | | ↑* | | ↓* | | | ↓** | | ↓** | ↓** | ↑ | | HYP↓** | | | | | |  |
| 1. **Glycyrrhizin arginine salt** | | |  |  |  |  |  |  |  |  |  |  |  |  |  |  |  |  |  |  |  |  |  |  |  |  |  |  |  |  |  |  |  |
| Zhang et al. 2018: | | |  |  |  |  |  |  |  |  |  |  |  |  |  |  |  |  |  |  |  |  |  |  |  |  |  |  |  |  |  |  |  |
| **Rat treatment/Marker** | **Cytokines** | **MDA** | **SOD** | | | | **GSH** | | **NADPH-oxidase** | | | **Cytoglobin** | | | | **Nrf-2 mRNA** | | **Keap-1** | | | **P38-MAPK** | | **NF-kB-p65** | **iNos** | **eNos** | | **COX-1** | | **α-SMA** | | **Other** | | |
| Low dose | TGF-β↓* immunof | NA | NA | | | | NA | | NA | | | NA | | | | NA | | NA | | | NA | | NA | NA | NA | | NA | | ↓ immunof+western blot | |  | | |
| High dose | TGF-β↓* immunof+western blot, TNF-α ↓* western blot | NA | NA | | | | NA | | NA | | | NA | | | | NA | | NA | | | NA | | ↓* | NA | NA | | NA | | ↓* immunof + western blot | | ↓* for MMP-2 and MMP-9 | | |
| 1. **Statins** | | |  |  |  |  |  |  |  |  |  |  |  |  |  |  |  |  |  |  |  |  |  |  |  |  |  |  |  |  |  |  |  |
| **Rat treatment/Marker** | **Cytokines** | **iNOS** | | | | **Collagen** | | | **α-SMA** | | | | | | **Other** | | | | | | | | |  |  |  |  |  |  |  |  |  |  |
| Tripathi et al. 2018 | |  |  |  |  |  |  |  |  |  |  |  |  |  |  |  |  |  |  |  |  |  |  |  |  |  |  |  |  |  |  |  |  |
| LPS (ACLF) | mRNA: IL-1β ↑*, IL-6 ↑ ↑*, TNF-α ↑*, IL-10 ↓ | ↑* (protein), ↑* (mRNA) | | | | NA | | | NA | | | | | | TLR-4 ↔, ICAM protein↑*,mRNA↑*, protein CD-68:↑ | | | | | | | | |  |  |  |  |  |  |  |  |  |  |
| Simvastatin | mRNA: IL-1β ↑*, IL-6 ↑*,**, TNF-α↔**, IL-10 ↓ | ↑ (protein), ↑*.** (mRNA) | | | | NA | | | NA | | | | | | TLR-4↔, ICAM protein↑ mRNA: ↑*,**, protein CD-68↓* | | | | | | | | |  |  |  |  |  |  |  |  |  |  |
| Meireles et al. 2017 | |  |  |  |  |  |  |  |  |  |  |  |  |  |  |  |  |  |  |  |  |  |  |  |  |  |  |  |  |  |  |  |  |
| BDL + hypovolemic shock and resuscitation | IL-1β ↑, IL-6 ↑ | NA | | | | NA | | | NA | | | | | |  | | | | | | | | |  |  |  |  |  |  |  |  |  |  |
| BDL + hypovolemic shock and resuscitation + Simvastatin | Normalized IL-1β and IL-6 | NA | | | | NA | | | NA | | | | | |  | | | | | | | | |  |  |  |  |  |  |  |  |  |  |
| Jang et al. 2018 | |  |  |  |  |  |  |  |  |  |  |  |  |  |  |  |  |  |  |  |  |  |  |  |  |  |  |  |  |  |  |  |  |
| TAA+Simvastatin | TGF-β ↓* | NA | | | | ↓ | | | ↓ | | | | | |  | | | | | | | | |  |  |  |  |  |  |  |  |  |  |
| TAA+Simvastatin + MSCs | TGF-β ↓* | NA | | | | ↓ | | | ↓ | | | | | |  | | | | | | | | |  |  |  |  |  |  |  |  |  |  |
| 1. **Lanifibranor** | |  |  |  |  |  |  |  |  |  |  |  |  |  |  |  |  |  |  |  |  |  |  |  |  |  |  |  |  |  |  |  |  |
| **Rat treatment/Marker** | **Cytokines** | **iNOS** | | | | **Collagen** | | | **α-SMA** | | | | | | **Other** | | | | | | | | |  |  |  |  |  |  |  |  |  |  |
| Boyer-Diaz et al. 2020 | |  |  |  |  |  |  |  |  |  |  |  |  |  |  |  |  |  |  |  |  |  |  |  |  |  |  |  |  |  |  |  |  |
| TAA + Lanifibranor | mRNA: IL-1β↓, IL-6↓*, TNF-α↓, IL-10↑ | ↓ | | | | NA | | | ↓* | | | | | | Arg↑, Mrc1↑ | | | | | | | | |  |  |  |  |  |  |  |  |  |  |
| 1. **WKYMVm** | |  |  |  |  |  |  |  |  |  |  |  |  |  |  |  |  |  |  |  |  |  |  |  |  |  |  |  |  |  |  |  |  |
| Jun et al. 2021 | |  |  |  |  |  |  |  |  |  |  |  |  |  |  |  |  |  |  |  |  |  |  |  |  |  |  |  |  |  |  |  |  |
| BDL+WKYMvm | IL-6↑* | NA | | | | ↓* | | | ↓* | | | | | | Gp130↑, STAT3↑*, VEGF↑, VEGFR1↑*, VEGFR2↑*, | | | | | | | | |  |  |  |  |  |  |  |  |  |  |

**Table Legends:**

**α-SMA:** α-smooth muscle actin, **ACLF**: acute on chronic liver failure; **Arg1**: arginase 1; **BDL:** bile duct ligation; **CCl4:** carbon tetrachloride; **c-fos:** *a proto oncogene;* **CHOP:** CCAAT/enhancer binding homologous protein; **CINC-1**: cytokine-induced neutrophil chemoattractant 1; **COX-2:** cyclooxygenase 2; **CTGF:** connective tissue growth factor; **eNOS:** endothelial nitric oxide synthase; **iNOS:** inducible nitric oxide synthase; **FSP-1**: fibroblast specific protein 1; **gp130:** glycoprotein 130; **GPx**: gluthathione peroxidase; **GR**: gluthatione reductase; **GSH:** glutathione reductase; **GRP78:** glucose-regulated protein 78; **HIF-1a:** hypoxia-inducible factor 1a; **HYP:** hydroxyproline; **Keap-1**: Kelch-like ECH-associated protein 1; **IL-1β:** interleukine 1β; **IL-6:** interleukine 6; **IL-8:** interleukine 8; **ICAM:** intercellular adhesion molecule; **LPS:** lipopolysaccharide; **MDA:** malondialdehyde; **MMP-2:** matrix metalloproteinase-2; **MMP-9:** matrix metalloproteinase-9; **Mrc1**: mannose receptor C-type 1, **MSCs**: mesenchymal stem cells; **NADPH-oxidase:** nicotinamide adenine dinucleotide phosphate oxidase; **NF-kB-p65:** nuclear factor-kB p65; **NOX-4**: nicotinamide adenine dinucleotide phosphate oxidase-4; **Nrf-2:** Nuclear factor erythroid 2-related factor 2; **P38-MAPK:** P38 mitogen activated protein kinases; **pErk:** endoplasmatic reticulum kinase; **PGE-2:** prostaglandine E-2; **Phospho-Akt:** *antibodies;* **Phospho-Smad2/3:** *antibodies;* **SMA:** smooth muscle actin; **Snail1:** *a gene;* **SOD:** superoxide dismutase; **TAA:** thioacetamide; **TGF-β:** transforming growth factor-β; **TLR4:** Toll-like receptor 4; **TNF-α:** tumor necrosis factor-α; **UPR:** unfolded protein response; **VEGF:** vascular endothelial growth factor; **WKYMVm**: *a hexapeptide*
